# Three multi-allelic gene pairs are responsible for self-sterility in the ascidian *Ciona intestinalis*

**DOI:** 10.1038/s41598-020-59147-4

**Published:** 2020-02-13

**Authors:** Hitoshi Sawada, Kazunori Yamamoto, Akira Yamaguchi, Lixy Yamada, Arata Higuchi, Haruhiko Nukaya, Masashi Fukuoka, Tetsushi Sakuma, Takashi Yamamoto, Yasunori Sasakura, Maki Shirae-Kurabayashi

**Affiliations:** 1 Sugashima Marine Biological Laboratory, Graduate School of Science, Nagoya University, Sugashima, Toba 517-0004 Japan; 2 Division of Integrated Sciences for Life, Graduate School of Integrated Sciences for Life, Hiroshima University, Hiroshima, 739-8526 Japan; 3 Shimoda Marine Research Center, University of Tsukuba, Shimoda, Shizuoka 415-0025 Japan

**Keywords:** Extracellular signalling molecules, Development

## Abstract

Many hermaphroditic organisms possess a self-incompatibility system to avoid inbreeding. Although the mechanisms of self-incompatibility in flowering plants are well known, little is known about the mechanisms of self-sterility in hermaphroditic marine invertebrates. Ascidians are hermaphroditic sessile marine invertebrates that release sperm and eggs into the surrounding seawater. Several species, including *Ciona intestinalis* type A *(Ciona robusta)*, exhibit strict self-sterility. In a previous study, we found that the candidate genes responsible for self-sterility in *Ciona* reside in chromosome 2q (locus A) and chromosome 7q (locus B). Two pairs of multi-allelic genes, named *s(sperm)-Themis-A* and *v(vitelline-coat)-Themis-A* in locus A and *s-Themis-B* and *v-Themis-B* in locus B, are responsible for self-sterility. In this study, we identified a third multi-allelic gene pair, *s-Themis-B2* and *v-Themis-B2*, within locus B that is also involved in this system. Genetic analysis revealed that the haplotypes of *s/v-Themis-A, s/v-Themis-B* and *s/v-Themis-B2* play essential roles in self-sterility. When three haplotypes were matched between *s-Themis* and *v-Themis*, fertilization never occurred even in nonself crossing. Interestingly, gene targeting of either *s/v-Themis-B/B2* or *s/v-Themis-A* by genome editing enabled self-fertilization. These results indicate that *s/v-Themis-A, -B* and *-B2* are *S-*determinant genes responsible for self-sterility in the ascidian *C. intestinalis* type A.

## Introduction

Most living organisms produce offspring by sexual reproduction to create genetic diversity in the next generation. However, hermaphroditic marine invertebrates such as ascidians (phylum Chordata, subphylum Tunicata) potentially allow self-fertilization. Although compound ascidians appear to release sperm and eggs at different times to avoid self-fertilization, several solitary ascidians are known to release sperm and eggs almost simultaneously into the surrounding seawater during the spawning season. However, even such ascidians show self-sterility^1^ or a preference for nonself-fertilization over self-fertilization^2^. In particular, *Halocynthia roretzi* and *Ciona intestinalis* type A (recently proposed to be renamed *Ciona robusta*^3^) exhibit strict self-sterility, although their mechanisms for prohibiting self-fertilization appear to be very different^4,5^.

In *H. roretzi*, HrVC70, a 70-kDa protein that is a main component of the vitelline coat (VC), appears to be responsible for self-sterility; HrVC70 attaches to the VC during oocyte maturation, wherein self-sterility is acquired, and HrVC70 can be specifically extracted from the isolated VC by 1–5 mM HCl, which is in agreement with the results showing that self-sterile eggs become self-fertile following short treatment with acidic seawater (pH 2.5–3.0). Importantly, nonself-sperm, rather than self-sperm, efficiently bound to HrVC70-immobilized beads, and HrVC70 from nonself-eggs more efficiently inhibited fertilization than did HrVC70 from self-eggs. In addition, HrVC70 is composed of 12 EGF-like repeats showing a polymorphism in restricted regions among individuals. These biological and biochemical data led us to propose that HrVC70 is a candidate protein involved in self-sterility in *H. roretzi* (see reviews^4,5^).

In contrast, in *Ciona intestinalis* type A, a HrVC70 orthologue, CiVC57, is present in the VC; however, it is not known whether CiVC57 is responsible for self-sterility, although CiVC57 appears to be involved in gamete interaction^5^. Thus, we attempted to identify *S (Self-sterility* or *Self-incompatibility)-*determinant gene(s) in *C. intestinalis* type A by a genetic approach. Thomas Hunt Morgan first studied the genetic mode of self-sterility in *Ciona* approximately a century ago (beginning in 1910)^6–9^. He reported that self-sterility is genetically controlled because artificially self-fertilized F1 siblings showed one-way or reciprocal sterility following crossing; this sterility was scarcely observed in natural crossing between nonself-fertilized siblings^8,9^. The mode of self-sterility was later studied by Murabe and Hoshi, who showed that at least two multi-allelic loci are involved in self-sterility^10^. Rosati and De Santis^11^ and Kawamura and his colleagues^12^ investigated the mechanisms of self-sterility in *Ciona*. These authors showed that self/nonself recognition in gamete interaction takes place on the VC and that nonself-sperm, but not self-sperm, are capable of binding tightly to the VC of glycerinated eggs^11,12^. However, the molecular mechanism of self/nonself recognition during fertilization is not known.

**Figure 1:**
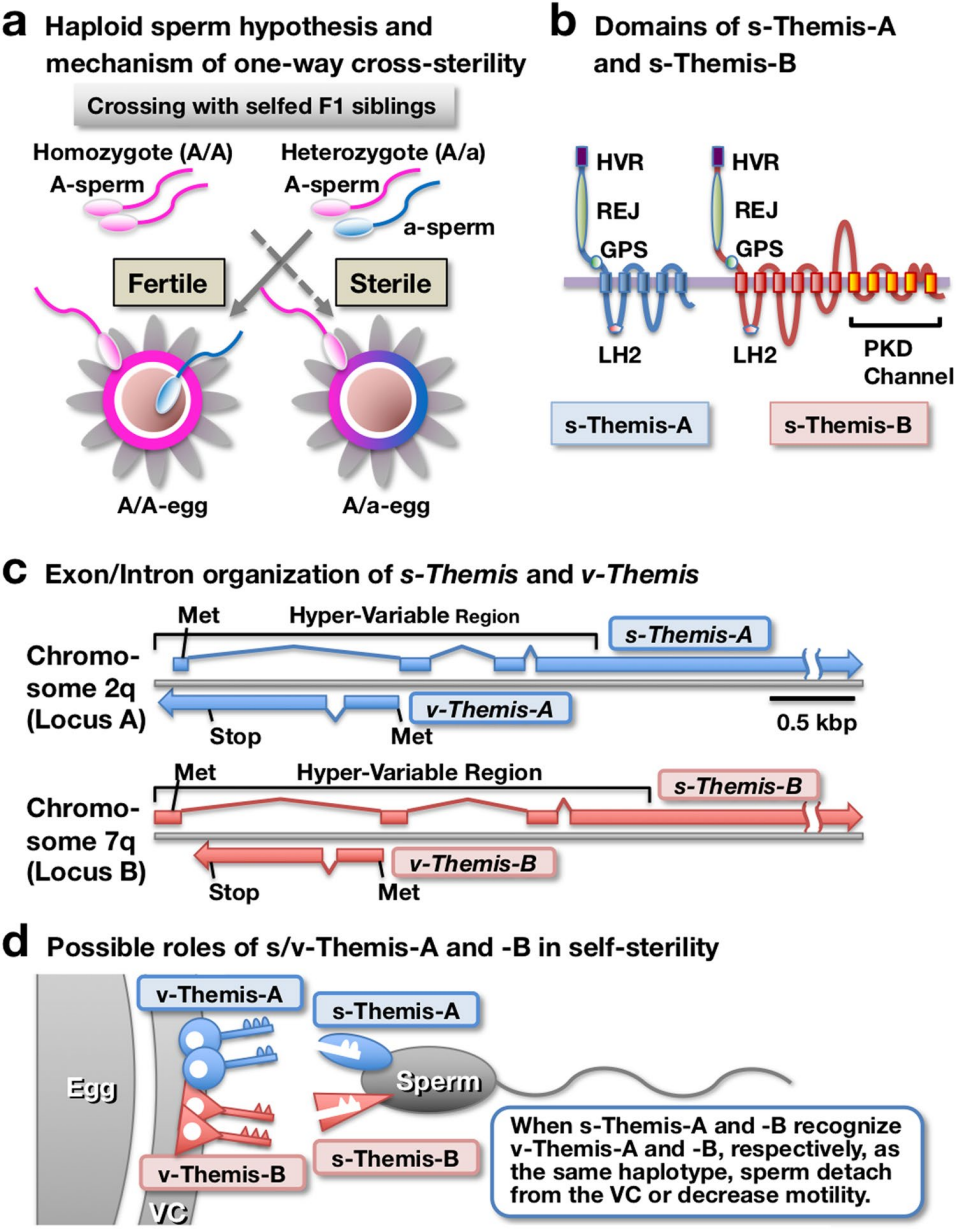
Candidate genes (*s/v-Themis-A* and *-B*) involved in self-sterility in *C. intestinalis* type A. (**a**) Haploid sperm hypothesis and one-way cross-sterility mechanism proposed by Morgan^8,9^. In selfed F1 siblings, there are two populations in heterozygous (A/a) sperm (A-sperm and a-sperm), either of which can fertilize homozygous eggs (A/A-eggs). In contrast, homozygous sperm (A-sperm or a-sperm) cannot fertilize heterozygous A/a-eggs because both receptors for A-sperm and a-sperm exist on the VC of A/a-eggs. Based on these criteria, candidate genes responsible for self-sterility were explored by positional cloning and proteomic analysis of the VC. (**b**) Transmembrane and other domains (HVR, hypervariable region; REJ, receptor for egg jelly; GPS, G-protein coupled receptor proteolysis site; and LH2, lipoxygenase homology 2) of s-Themis-A and s-Themis-B are illustrated. Note that only B-type s-Themis contains a C-terminal PKD channel. (**c**) *s-Themis-A* and *v-Themis-A* genes reside in locus A of chromosome 2q, and *s-Themis-B* and *v-Themis-B* genes reside in locus B of chromosome 7q. In both cases, the *v-Themis* gene is located in the first intron of the *s-Themis* gene but is transcribed in the opposite direction. (**d**) According to Morgan’s hypothesis, it is thought that *s-Themis-A* and *-B* genes have haploid expression, while *v-Themis-A* and *-B* genes have diploid expression. When sperm s-Themis-A and s-Themis-B recognize one allele of v-Themis-A and v-Themis-B, respectively, as the same allele haplotypes, the spermatozoon detaches from the VC or decreases motility to prohibit self-fertilization.

We have explored candidate genes responsible for self-sterility in *C. intestinalis* type A by positional cloning and proteomic analysis^13^. To explain one-way cross-sterility, Morgan proposed a “haploid sperm hypothesis”, according to which self-sterility is achieved by haploid expression of the *S-*determinant gene in spermatozoa and diploid expression in eggs (Fig. 1a, see also refs. ^8,9^). According to his hypothesis, there are two populations in heterozygous (A/a) sperm (A-sperm and a-sperm), either of which can fertilize homozygous eggs because A/A-eggs (or a/a-eggs) are recognized by a-sperm (or A-sperm) as nonself eggs (Fig. 1a). In contrast, a heterozygous A/a-egg cannot be fertilized by homozygous sperm (A-sperm or a-sperm) because both receptors for A-sperm and a-sperm exist on the VC of A/a-eggs, resulting in blocked fertilization (Fig. 1a). Therefore, if a one-way cross-sterile combination was observed, the *S*-determinant gene should be heterozygous in “males” and homozygous in “females” (Fig. 1a). Based on these criteria, Harada *et al*.^13^ used PCR to determine whether approximately 70 genes that are suspected of being responsible for self-sterility were homozygous or heterozygous.

With this approach, Harada *et al*.^13^ revealed that *S-*determinant genes reside in two multi-allelic loci: locus A (chromosomes 2q) and locus B (chromosome 7q). Each locus contained a pair of similar genes despite there being no overall synteny, *i.e*., fibrinogen-like proteins expressed in the VC, designated *v(vitelline coat)-Themis-A* (in locus A) and *v-Themis-B* (in locus B), and sperm polycystin 1-like proteins named *s(sperm)-Themis-A* (in locus A) and *s-Themis-B* (in locus B) (Fig. 1c). The entire region of v-Themis-A and v-Themis-B and a hyper-variable region (HVR) of s-Themis-A and s-Themis-B showed polymorphisms among individuals^13^. Proteomic analysis of the VC revealed the existence of v-Themis-A and v-Themis-B on the VC^13,14^. Both *v-Themis-A* and *v-Themis-B* genes were encoded in the first intron of *s-Themis-A* and *s-Themis-B* genes, respectively, but were transcribed in the opposite direction from *s-Themis* genes (Fig. 1c). This finding suggests that these gene pairs (haplotypes) are hardly segregated by recombination during meiosis^13^. Genetic analysis revealed that self-sterility in *C. intestinalis* type A is governed by the self-recognition system. According to Morgan’s hypothesis, self-sterility appears to be achieved by haploid expression of *s-Themis-A* and *s-Themis-B* in spermatozoa and by diploid expression of *v-Themis-A* and *v-Themis-B* in the VC^13^ (Fig. 1d). We proposed that a spermatozoon recognizes the VC of the egg as self-egg when sperm s-Themis-A and s-Themis-B both recognize the same haplotype v-Themis-A and v-Themis-B, respectively^13^ (Fig. 1d). s-Themis-B contains a C-terminal Ca^2+^-permeable cation channel (polycystic kidney diseases (PKD) channel) domain in addition to the domains shared with s-Themis-A, including HVR, receptor for egg jelly (REJ), G protein-coupled receptor proteolysis site (GPS), and lipoxygenase homology 2 (LH2) domains^13^ (Fig. 1b). We previously reported that a spermatozoon undergoes drastic Ca^2+^ influx upon sperm binding to the VC of self-eggs (but not to the VC of nonself-eggs), which is followed by detachment from the VC or by the sperm entering a quiescent state on the VC^15^. However, it is still unclear for the reasons stated below whether *s/v-Themis-A* and *s/v-Themis-B* gene pairs alone are sufficient for execution of self-sterility in *C. intestinalis* type A.

We previously reported that one additional B-type *s/v-Themis* gene pair may exist near the *s/v-Themis-B* gene in locus B, since three alleles of B-type v-Themis proteins were detected in the VC by mass spectrometry^13,14^; further, one additional *s/v-Themis-B* gene pair has been proposed to exist in locus B on the basis of the Ghost database (*C. intestinalis* type A genome database)^16^. In addition, it was recently reported that only the *s/v-Themis-B* gene region showed heterogeneity in an inbred strain and that the copy number of *s/v-Themis-B* gene pairs is variable among individuals^17^. These unexpected results led us to further investigate the gene structures and their roles in the self-sterility of B-type *s/v-Themis*. Since the DNA sequence around *s/v-Themis-B* has not yet been completely determined, we first cloned and sequenced the genomic DNA in locus B. We identified a new gene pair, designated *s-Themis-B2* and *v-Themis-B2*, which resides approximately 70 kbp away from the *s/v-Themis-B* gene pair. We also showed that three gene pairs, *s/v-Themis-A, -B* and *-B2*, are *S*-determinant genes responsible for self-sterility in *C. intestinalis* type A.

## Results

### Heterogeneity in the *s/v-Themis-B* gene region

To determine the DNA sequence around *s/v-Themis-B*, we isolated and sequenced a 35-Mb genomic DNA clone containing *s/v-Themis-B* from the BAC library of *C. intestinalis* type A genomic DNA, and we compared the genomic DNA sequences in the Ghost database^16^ and the JGI database^18^. Our newly determined BAC clone sequence, designated JP1 haplotype (or B-6 haplotype), was compared with DNA sequences in the two databases by dot matrix plot analysis (Fig. S1). The results showed that the DNA sequence in the JGI database coincided well with the sequence of JP1 (Fig. S1b). On the other hand, there are two gaps in the Ghost database^16^, in which one truncated sequence of the *s/v-Themis-B* gene pair containing HVR was inserted upstream of the *s-Themis-B* gene (Fig. S1a). These results suggest two possibilities. First, the Ghost database has an error in the DNA sequence due to possible scaffold misassembly. Second, some individuals have an additional partial sequence of the *s/v-Themis-B* gene pair upstream of the *s-Themis-B* gene.

By careful inspection, we recently noticed that one or more additional HVR(s) were located at the *s-Themis-B* locus in several individuals, similar to what was observed in the Ghost database. However, we could not detect more than two types of mRNAs from *s-Themis-B* genes by 5′-RACE, indicating the possibility that few or no truncated *s -Themis-B* genes are transcribed. These results, together with the fact that the truncated *s-Themis-B* gene has no C-terminal cation channel domain, suggest that these truncated genes are non-functional. Recently, the Ghost database was updated based on genome assembly for an inbred line of this animal^19^.

### Identification of a pair of *s-Themis-B2* and *v-Themis-B2* genes

**Figure 2:**
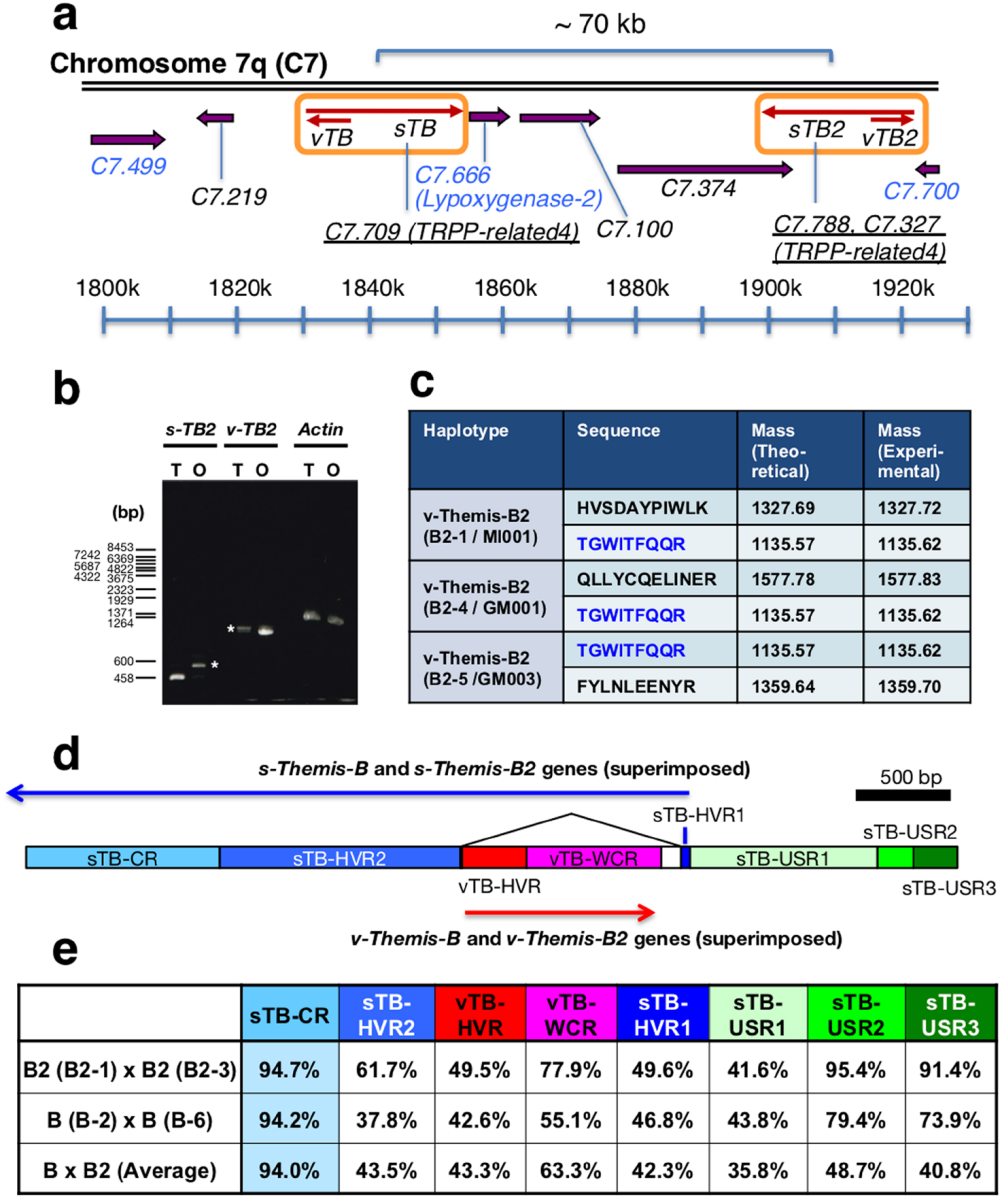
Genomic localization and mRNA expression of *s/v-Themis-B2* genes. (**a**) *s/v-Themis*-*B2* genes are located on chromosome 7q, with a distance of approximately 70 kbp from *s/v-Themis-B*. Positions of respective gene model IDs and transcriptional directions are indicated. PCR primers used for specific amplification of *s-Themis-B* and *s-Themis-B2* were designed on the basis of the sequences of gene models indicated by blue letters (for details, see Materials and Methods and Table S2). Annotations and domains of the respective gene models are shown online (http://www.aniseed.cnrs.fr). The *s/v-Themis-B* and *s/v-Themis-B2* genes are indicated by red arrows and orange open boxes, whereas the other genes are indicated by purple arrows. (**b**) *s-Themis-B2 (sTB2)* and *v-Themis-B2 (vTB2)* are transcribed in the testis (T) and ovary (O), respectively, as revealed by RT-PCR. Asterisks indicate genomic DNA of *s-Themis-B2* and *v-Themis-B2*. (**c**) Proteomic analysis of VC proteins digested with trypsin. v-Themis-B2-specific fragments were detected by mass spectrometry using a MASCOT search engine. Peptides with scores higher than 20 were accepted as identified. The mass values calculated from the respective amino acid sequences were indicated as “Mass (Theoretical)”, and the mass values obtained by mass spectrometry were indicated as “Mass (Experimental)”. An identified fragment indicated by blue had the same sequence as that in a relatively conserved region of v-Themis-B2. (**d**) Determined nucleotide sequences of two *s/v-Themis-B2* alleles (B2-1 (=MI001) and B2-3 (=MI002)) and two *s/v-Themis-B* alleles (B-2 (=JGI) and B-6 (=JP1)) were aligned (Fig. S2), and the following regions are indicated by the following colours: sTB-CR (*s-Themis-B* conserved region: sky blue), sTB-HVR2 (*s-Themis-B* hypervariable region 2: blue), vTB-HVR (*v-Themis-B* hypervariable region: red), vTB-WCR (*v-Themis-B* weakly conserved region: magenta), sTB-HVR1 (*s-Themis-B* hypervariable region 1: dark blue), sTB-USR1 (*s-Themis-B* upstream region 1: pale green), sTB-USR2 (*s-Themis-B* upstream region 2: green), and sTB-USR3 (*s-Themis-B* upstream region 3: dark green). Transcriptional directions of *s-Themis-B* and *-B2* (blue) and *v-Themis-B and -B2* (red) are indicated by arrows. (**e**) Identities shared between the B2 allele (B2-1) and B2 allele (B2-3), between the B allele (B-2) and B allele (B-6), and between the B and B2 alleles (average) are indicated by percentage identity. Note that sTB-CR showed very high identity between the B and B2 regions. Respective regions are indicated by the same colours. The abbreviations used are as follows: *C7*, chromosome 7; *sTB, s-Themis-B; sTB2, s-Themis-B2; TRPP, transient receptor potential polycystic; vTB, v-Themis-B; vTB2, v-Themis-B2;* O, ovary; T, testis.

Next, we searched for another putative B-type *s/v-Themis* gene pair in locus B by primer walking, and we identified a novel *s/v-Themis* gene pair, designated *s/v-Themis-B2*. These genes were approximately 70 kbp away from the locus of *s/v-Themis-B* (Fig. 2a). The results showed that *s-Themis-B2* and *v-Themis-B2* genes were transcribed in the testis and ovary, respectively (Fig. 2b), and that they are highly polymorphic multi-allelic genes (Figs. 2d,e, S2). The DNA sequences of *s-Themis-B* and *s-Themis-B2* were almost identical except for N-terminal HVR (Figs. 2d,e, S2). Upstream regions (USR1-USR3) of the *s-Themis-B/B2* genes also showed diversity in DNA sequences (Fig. 2e). These results imply that *s/v-Themis-B* and *s/v-Themis-B2* gene pairs were duplicated during evolution. Trypsin-digested fragments of v-Themis-B2 alleles (Fig. 2c) as well as v-Themis-A alleles (Fig. S3) and v-Themis-B alleles (Fig. S4) were detected in the VC by mass spectrometry. We also attempted to identify s-Themis-A, -B and -B2 proteins by mass spectrometry but have not yet succeeded probably because multi-transmembrane-domain-containing high molecular weight proteins, such as s-Themis, may be difficult to solubilize from sperm membranes with regular SDS-sample buffer followed by heat denaturation.

### Comparison between *s/v-Themis-B* and *s/v-Themis-B2* haplotypes

Ten alleles (B-1, 2, 3, 4, 5, 6, 7, 8, 10, and 11) for *s/v-Themis-B* and 7 alleles (B2-1, 2, 3, 4, 5, 6, and 8) for *s/v-Themis-B2* have been identified (see Table S1). We compared these 17 alleles with 12 alleles (designated A - L) identified by Satou *et al*.^17^ and examined whether each allele matched with the *s/v-Themis-B* allele or the *s/v-Themis-B2* allele by amplifying the DNA of the respective HVR. As a result, 7 alleles reported by Satou *et al*.^17^ matched our *s/v-Themis-B* and *s/v-Themis-B2* alleles (see Table S1). Importantly, 22 alleles belonged to either *s/v-Themis-B* or *s/v-Themis-B2* but not to both. We noticed that their primers could amplify not only HVRs of complete forms of *s/v-Themis B* and *s/v-Themis-B2* but also the HVR of the truncated form of *s/v-Themis-B*, which is located upstream of *s-Themis-B* as in the Ghost database. There may be a special gene sequence or chromosome structure around this region that stimulates DNA duplication and/or recombination.

### *s/v-Themis-A, -B*, and *-B2* are *S*-determinant genes as revealed by genetic analysis

**Figure 3:**
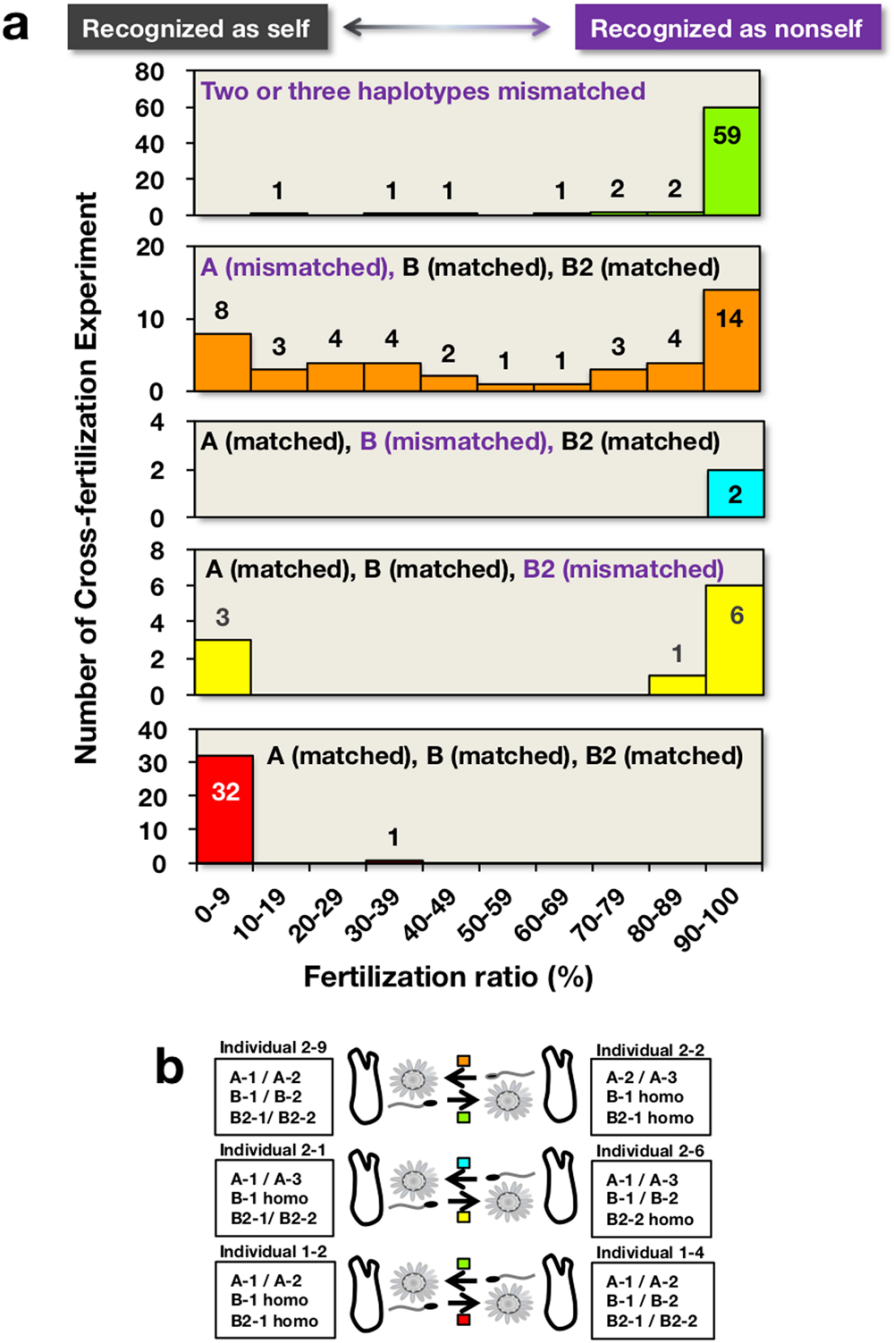
Fertilization ratios in out-crossing with selfed F1 and F2 siblings. (**a**) Fertilization ratios were determined by scoring the numbers of 2- or 4-cell stage embryos and unfertilized eggs 1 h after insemination. The results are expressed by a histogram showing the number of trials of cross-fertilization (ordinate) versus the fertilization ratio (%, abscissa) under the following combinations of conditions: top panel, two or three haplotypes mismatched (green); second panel, B and B2, but not A, haplotypes matched (orange); third panel, A and B2, but not B, haplotypes matched (blue), fourth panel, A and B, but not B2, haplotypes matched (yellow); fifth panel, all three haplotypes (A, B and B2) matched (red). (**b**) Schematic drawing of gamete recognition patterns between different alleles and the same alleles. *s-Themis-A, -B* and *-B2* have haploid expression in spermatozoa, whereas *v-Themis-A, -B*, and *-B2* have diploid expression on the VC. When the sperm s-Themis protein recognizes one of the two alleles of v-Themis as the same haplotype, the s-Themis protein appears to recognize the v-Themis as self. Boxed colours indicate the categories indicated in (**a**). “Trial number” indicates the results of fertilization experiments summarized in Fig. S5.

To determine whether *s/v-Themis-A, -B*, and *-B2* are *S*-determinant genes, we performed genetic analysis by cross-fertilizing selfed F1 and F2 siblings, whose *s/v-Themis-A, -B*, and *-B2* alleles had been identified using allele-specific PCR primers. According to the “haploid sperm hypothesis”^8,9^, there must be haploid expression in *s-Themis-A, -B*, and *-B2* alleles and diploid expression in *v-Themis-A, -B*, and *-B2* alleles. When sperm s-Themis-A, -B, and -B2 recognize either of two alleles of v-Themis-A, -B, and -B2 as the same haplotype, respectively, fertilization is thought to be blocked (Fig. 1). Fertilization ratios (%) between selfed F1 and F2 siblings are summarized in Fig. 3a, and the raw data are shown in Fig. S5. Five combination patterns are indicated by different colours in Fig. 3a: two or three haplotypes mismatched (green); B and B2, but not A, haplotypes matched (orange); A and B2, but not B, haplotypes matched (blue); A and B, but not B2, haplotypes matched (yellow); and three (A, B, and B2) haplotypes matched (red). For instance, in crosses between individuals 2–9 and 2–2 (Fig. 3b top cross; see also Fig. S5 Trial 2), only the A haplotype did not match using the right-individual sperm and the left-individual eggs (orange), whereas three haplotypes were mismatched using the left sperm and the right eggs (green). Similarly, in crosses between individuals 2–1 and 2–6 (Fig. 3b middle), only the B haplotype was mismatched using the right sperm and the left eggs (blue), whereas only the B2 haplotype was mismatched using the left sperm and the right eggs (yellow). In crosses between individuals 1–2 and 1–4 (Fig. 3b bottom), B and B2 haplotypes were mismatched using the right sperm and the left eggs (green), whereas three haplotypes matched using the left sperm and the right eggs (red).

As summarized in Fig. 3a, it is evident that nonself-fertilization never occurred when all three haplotypes in *s/v-Themis-A, -B and -B2* matched (see red columns in Figs. 3a and S5). These results unambiguously demonstrated that the three *s/v-Themis* gene pairs are responsible for self-sterility. When two or three alleles were mismatched haplotypes, sperm overwhelmingly recognized the VC as nonself, allowing fertilization (see green columns in Fig. 3a). However, in the case of a single haplotype being mismatched in A, B, or B2, the sperm recognized the VC as nonself in most cases, but there were some exceptions (see Fig. 3a). Even if the A and B haplotypes matched, the sperm recognized the VC as nonself and fertilized the eggs when the B2 haplotype was mismatched, but there were some exceptional cases (see yellow columns in Fig. 3a). These results indicate that the *s/v-Themis-B2* gene pair is involved in self-sterility, although there were three exceptional cases, among which two cases were explained by the fact that B2-3 (MI002) haplotypes were not functional, as discussed later. These allele analysis experiments clearly demonstrated that *s/v-Themis-A, -B*, and *-B2* are *S*-determinants that play a pivotal role in self-sterility in *C. intestinalis* type A.

### *s/v-Themis-A, -B*, and *-B2* are essential for self-sterility

**Figure 4:**
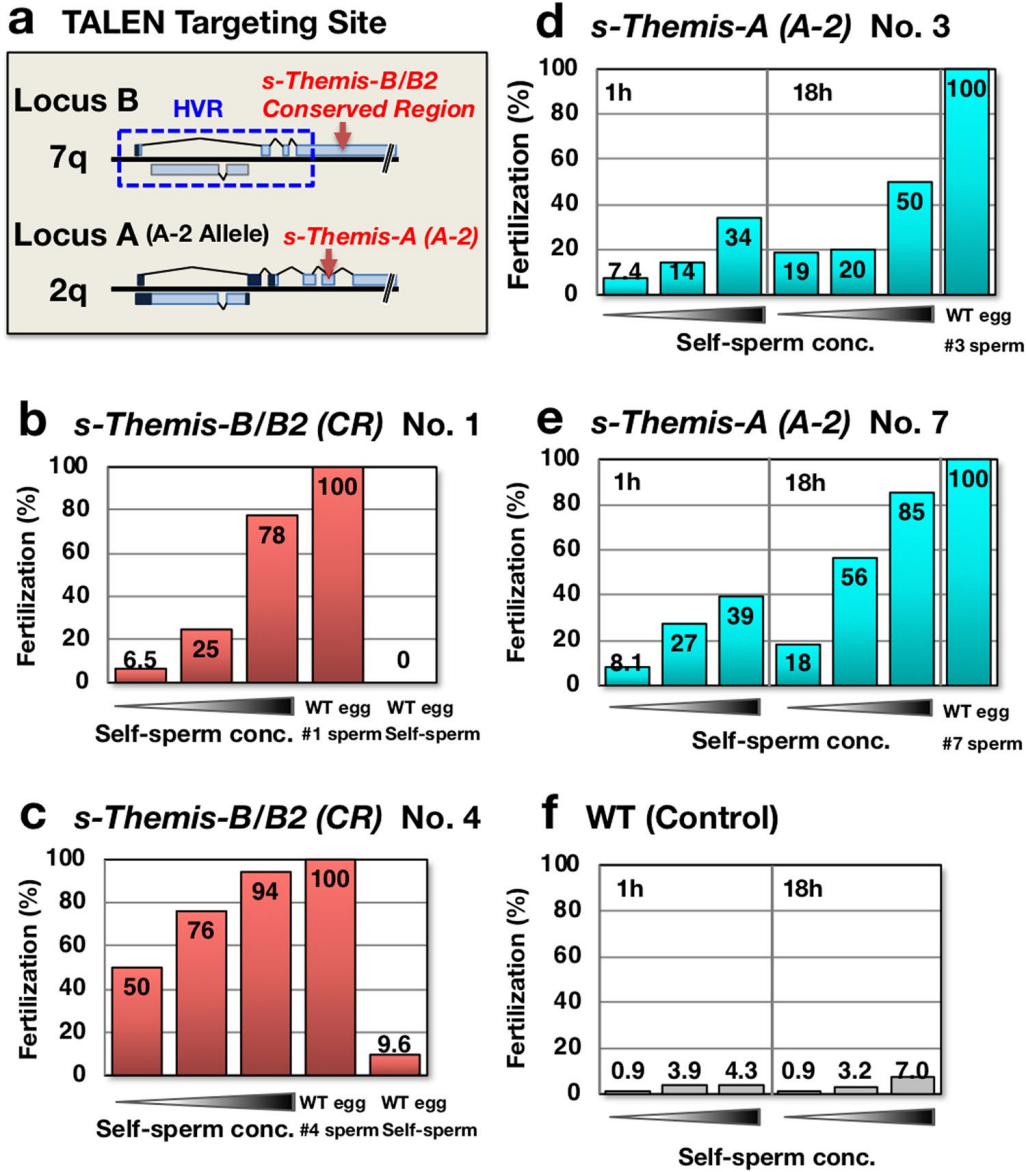
Self- and nonself-fertilization experiments using TALEN-injected animals. (**a**) The TALEN targeting sites were located in a conserved region (downstream of the HVR of *s-Themis* and upstream of *v-Themis*) of *s-Themis-B/B2* (**b,c**) and in the indicated region of the A-2 (G7) allele of *s-Themis-A* (**d,e**). After injection of TALEN mRNA or plasmid into fertilized eggs by electroporation, the embryos were grown to produce adults. Proper induction of target mutagenesis in the experimental animals was confirmed by Cel-1 assays (Fig. S7). Sperm and eggs were obtained from these animals and were then subjected to self- and nonself-fertilization experiments. Fertilization ratios were determined 1 h after insemination using serially diluted sperm (sperm concentration increases from left to right.). The numbers in the panels indicate the experiments using different individuals. Two representative results are shown for each experiment. Untreated wild-type eggs were inseminated with respective targeted sperm at the lowest concentration or with self-sperm from a wild-type individual. Representative results for the self-fertilization ratio in a wild-type individual using the same serial dilution of sperm are shown in (**f**).

To investigate whether these genes are essential for self-sterility, we carried out gene targeting of *s-Themis-A* and *s-Themis-B/B2* using TALEN (transcription activator-like effector nuclease). Fertilized eggs of individuals with known alleles were subjected to electroporation with TALEN plasmids or mRNAs. After electroporation, embryos were cultured for 3 months in the laboratory, and gametes were collected from sexually mature individuals. As shown in Fig. 4, the self-fertilization ratio overwhelmingly increased between sperm and eggs from *s-Themis-B/B2 CR* (conserved region)-targeted individuals. However, when the *s-Themis-A* (allele A-2) gene was targeted, self-fertilization was allowed but required a longer time (Fig. 4). These results indicate that both *s/v-Themis-A* and *s/v-Themis-B/B2* are essential for self-sterility in *C. intestinalis* type A.

## Discussion

**Figure 5:**
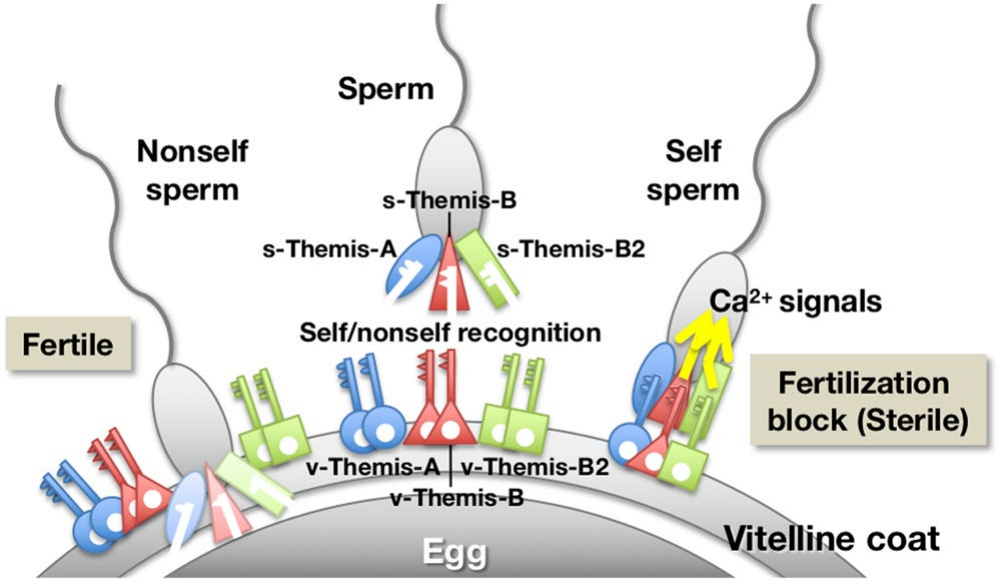
Working hypothesis on the roles of s/v-Themis-A, -B, and -B2 in self/nonself recognition during fertilization. Diploid expression of *v-Themis-A, -B* and *-B2* and haploid expression of *s-Themis-A, -B* and *-B2* allow one-way sterility. When three haplotypes are matched, Ca^2+^ influx occurs, resulting in the detachment of sperm from the VC or resulting in the sperm entering a quiescent sate on the VC, which prohibits fertilization.

In the present study, we demonstrated for the first time that three pairs of *s-Themis* and *v-Themis* genes, that is, *s/v-Themis-A, s/v-Themis-B* and *s/v-Themis-B2*, are responsible for self-sterility in *Ciona intestinalis* type A and that these gene pairs are *S*-determinant genes (Fig. 5).

Several haplotypes of s/v-Themis-A may not effectively function as self-recognition proteins because some crossings between individuals mismatched in A haplotypes (orange) showed a small or null fertilization ratio (Figs. 3a and S5). On the other hand, crossing between individuals that had matching A and B, but not B2, haplotypes (yellow) or matching A and B2, but not B, haplotypes (blue) showed fertilization in an all-or-none fashion. These results imply that A haplotypes may weakly participate in self-sterility. s-Themis-B/B2, rather than s-Themis-A, may play an important role in self-sterility since only B-type s-Themis contains a Ca^2+^-permeable cation channel domain and since Ca^2+^ influx occurs upon a self-recognition response^15^. By analogy of the TRP-type cation channel, it is plausible that a hetero-tetramer of s-Themis-B and s-Themis-B2 may construct a single Ca^2+^ channel^20^. s-Themis-A might indirectly support the functions of the s-Themis-B/B2 Ca^2+^ channel.

Concerning *s/v-Themis-B2*, it is notable that a certain haplotype in *s/v-Themis-B2* may not function as an *S*-determinant. Among the three exceptional cases in *s/v-Themis-B2*, in two exceptional cases shown in trial 1 (specifically, between individual-1 sperm and individual-2 eggs and between individual-1 sperm and invidual-4 eggs, see the yellow left column of Figs. 3a and S5 (Trial 1)), we found that the sequence of the B2-3 (MI002) haplotype possesses 20 termination codons in the *s-Themis-B2* cDNA ORF and 9 termination codons in the *v-Themis-B2* cDNA ORF (Fig. S6). In addition, the gene product of B2-3 allelic *v-Themis-B2* was not detected in the VC by mass spectrometry. These results suggest that *s/v-Themis-B2* (B2-3 allele) genes are non-functionable pseudogenes.

In the genome editing experiments, the self-fertilization ratio drastically increased between sperm and eggs from *s-Themis-B/B2 CR*-targeted individuals. In contrast, when the *s-Themis-A* (allele A-2) gene was targeted, self-fertilization was observed but required a longer time (Fig. 4). These results support our hypothesis that *s-Themis-B/B2* may play more important roles than *s-Themis-A* in self-sterility. Mutations of the *s-Themis-B/B2* and *s-Themis-A* genes in tadpole larvae from electroporated embryos were confirmed by a Cel-1 nuclease assay (Fig. S7). However, unexpectedly, most of the tadpole larvae derived from self-fertilized embryos from genome-edited adults showed in-frame mutations or no deletion/insertion at the targeting site (see Fig. S8). Although we do not have any evidence to explain this apparent discrepancy, it seems possible that *s-Themis* genes may be expressed in somatic (non-germ) cells in a haploid manner, which is different from how they are expressed in germ cells (illustrated in Fig. S9b). In flowering plants, there are two types of self-incompatibility systems: “gametophytic” and “sporophytic” self-incompatibilities^21,22^ (Fig. S9a,b). In the gametophytic system, *S*-determinant genes are expressed in haploid pollen (see Fig. S9a), in which impaired genes will be transmitted to the next generation. In contrast, in the sporophytic system, *S*-determinant genes are expressed in the diploid anther tapetum surrounding the pollen (see Fig. S9b). In this case, DNA mutations will not be transmitted to the next generation (Fig. S9b). It is well known that in mammals, sperm surface proteins are not necessarily expressed by germ cells. For instance, a cysteine-rich secretory protein (DE) is known to be expressed in the epididymis and to be transferred to the sperm cell surface via the epididymosome^23^, and CD9, an oocyte tetraspanin membrane protein, has been reported to be released from the egg surface as an exosome and fused with the sperm surface, which is a process that is essential for sperm fusion with an egg^24^.

It is notable that the mechanism of self-sterility in *Ciona* is similar to the self-incompatibility system in flowering plants, in which *S*-determinant gene pairs are known to be family specific^21,22^. Brassicaceae and Papaveraceae have self-recognition systems: the former utilizes pollen SP11/SCR and stigmatic SRK (S-locus receptor kinase), and the latter utilizes pollen PrpP and stigmatic PrpS^21,22^. On the other hand, Solanaceae and Rosaceae utilize nonself-recognition systems, where stigmatic *S*-RNase and pollen tube SLF (S-locus F-box protein), a ubiquitin ligase involved in the degradation of nonself-*S*-RNase that has entered a pollen tube, are nonself-recognition partners. From this viewpoint, it is not surprising that *Ciona* (order Phlebobranch) and *Halocynthia* (order Stolidobranch) utilize different *S*-determinant genes. It is not known whether *S*-determinant genes are genus-, family-, or order-specific. It also remains elusive whether s/v-Themis play a key role in Stolidobranch ascidians, although *s/v-Themis* genes are present in the genome database of *Halocynthia*.

In any case, it should be emphasized that there are two similar points in animal and plant self-incompatibility (or self-sterility) systems: one is that male and female *S*-determinant genes are highly polymorphic and tightly linked in the same or contiguous loci, and the other is that male and female *S*-determinants are a single haplotype that make up a recognition partner. In particular, the self-incompatibility system in Papaveraceae is very similar to the self-sterility system in *Ciona intestinalis* type A: calcium influx takes place, resulting in apoptosis in pollen to prohibit self-fertilization, after pollen PrpP recognizes the stigmatic PrsP as self^25^, while calcium influx takes place in *C. intestinalis* type A sperm after sperm binding to the VC of self-eggs^15^.

Our results provide new insights into the molecular mechanism of self-sterility in ascidians, which is very similar to the mechanisms of self-incompatibility in flowering plants. This is a good example of convergent evolution between plants and animals^26^. On the other hand, it has been reported that human sperm PKDREJ, a candidate sperm-born receptor^27^ and a homologue of ascidian sperm s-Themis-B/B2, exhibits amino acid polymorphisms among 14 primates and 48 human individuals^27^, and it has been proposed to have evolved by positive selection^28^. Therefore, it is interesting to speculate that allogeneic polymorphisms in PKDREJ might be related to the efficiency of human fertility, similar to how s-Themis-B/B2 functions in ascidians.

## Materials and Methods

### Animals, gametes and culture conditions of embryos

The ascidians, *Ciona intestinalis* type A (recently proposed to be renamed *Ciona robusta*), that were used in this study were collected in Gamagori, Mikawa Bay, and some of the ascidians were supplied by National BioResource Project (NBRP) from Tokyo and Maizuru Bay. Spermatozoa and eggs were collected as described previously^13,15,29^. Fertilization and embryo culture were carried out as described previously^13^. To determine the genotype of the animals, DNA was extracted from the testis, and direct sequencing of the HVRs of *s/v-Themis-A, -B*, and *-B2* was carried out using primers listed in Table S2(c). Hatched tadpole larvae were attached to a Petri dish filled with seawater and were allowed to undergo metamorphosis. After attachment, larvae or juveniles were cultured in a small aquarium filled with gently circulating seawater. The animals were fed, and seawater was replaced three times per week. Discarded seawater was sterilized with 1% hydrogen peroxide.

### DNA sequence around the *s/v-Themis-B* region and identification of *s/v-Themis-B2*

The DNA sequence in locus B was searched for with two *C. intestinalis* type A genome databases: the Ghost database (Kyoto Univ.; http://ghost.zool.kyoto-u.ac.jp/cgi-bin/gb2/gbrowse/kh/)^16,19,30^, which is linked to the Ascidian Network for *In Situ* Expression and Embryological Data (ANISEED) containing 13 ascidian databases (http://www.aniseed.cnrs.fr)^31^, and the JGI (DOE Joint Genome Institute) database (http://genome.jgi-psf.org/Cioin2/Cioin2.home.html)^18^.

### Nucleotide sequence determination of genomic DNA of *s/v-Themis-B* and *-B2*

PCR was carried out using a BAC DNA clone (GECi44_m19)^32^ encoding *s/v-Themis-B* as a template and P1(TBg) and P2(TBg) primers (for primer sequences, see Table S2(a)), corresponding to the upstream (KH.C7.499.v1.R.ND1-1 (Fig. 2a)) and downstream (KH.C7.666.v1.SL2-1) regions of *s/v-Themis-B* genes. Nucleotide sequences of PCR products were determined by primer walking as described previously^32^. Using genomic DNA isolated from several individuals as a template, PCR was carried out to amplify the 4.5-kbp region containing the HVR of *s/v-Themis-B2* by using primers P3(TBg) and P4(TBg), which correspond to the conserved region of *s-Themis-B2* and to the KH.C7.700.v1.C.ND1-1 that is located near *s/v-Themis-B2*. The amplified genomic DNA fragments were used as a template, and DNA sequencing was carried out by primer walking.

### Isolation and nucleotide sequence determination of *s/v-Themis-B cDNA*

To identify haplotypes, 5′-RACE was carried out by using testis cDNA as a template and primer P1 corresponding to the conserved region of *s-Themis-B*, which resulted in amplification of the 5′-region of *s-Themis-B*. Next, using the reverse primer P1(sTBc) (conserved region of *s-Themis-B*) and haplotype-specific forward primers (P2(sTBc) - P6(sTBc)) located in the first exon of *s-Themis-B*, PCRs were carried out for the region around *v-Themis-B* using genomic DNA as a template. Since there are one or more exons in *v-Themis-B, v-Themis-B* sequences were determined by using ovary cDNA as a template and two primers P7 and P12 that corresponded to the first and second exons, respectively (Table S2(b)).

### Dot matrix plot analysis

DNA dot matrix plot analysis was carried out according to DNA DotPlot v1.0 (http://genomics.cribi.unipd.it/DNA_DotPlot)^33^.

### Expression of *v-Themis-A, -B* and *-B2*

Using the sequences of cDNAs from all of the alleles of *v-Themis-A, B, B2* and genomic DNA, a database was constructed, and the entire sequences were subjected to MASCOT analysis by mass spectrometry^14^.

### Targeted mutagenesis of *s/v-Themis-A* and *s/v-Themis-B/B2* by TALEN

TALEN target sites within the *s-Themis-A* allele G7-specific region and *s-Themis-B/B2* conserved region genes were identified using the TAL Effector Nucleotide Targeter (TALE-NT) 2.0 program and GenomeScanZFN to avoid off-target effects. TALEN was constructed using a *Golden Gate TALEN* and TAL Effector Kit 2.0^34–39^. The TALEN mRNA was synthesized with mMASSAGE mMACHINE T3 (Ambion) and a Poly(A) Tailing kit (Ambion) using pHTB-Ci-s-Themis-A(A-2) and pHTB-Ci-s-Themis-B/B2(CR) plasmids^36,39^ as templates after treatment with XhoI. We also constructed TALENs targeting the genes s-Themis-A(A-2) and s-Themis-B/B2(CR) by inserting them into Ci-EF1α TALEN expression vectors^38,39^.

The VCs of *C. intestinalis* type A eggs were removed by gentle pipetting after two additions of 5 ml of filtered seawater (FSW) containing 0.2 g of sodium thioglycolate and 0.02 g of actinase E to an equal volume of egg suspension. After the VC had been removed by this treatment, the VC-free eggs were gently washed with FSW 3 times. The eggs were placed on a gelatine-coated Petri dish filled with FSW and were then inseminated. After 20 min, the eggs were washed twice with 5 ml of FSW containing 0.7 M mannitol and were put into a cuvette for electroporation. TALEN mRNA (60 µg) and TALEN expression vectors (pEF1a > TALENs) (60 µg) were added to the egg suspension and subjected to electroporation with a Gene Pulser Xcell electroporation system (BIO-RAD). The eggs were transferred to a new gelatine-coated Petri dish filled with seawater, washed with FSW 3 times, and then kept at 16–18 °C until the hatched larval stage.

### Cel-I assay

DNA was isolated from hatched larvae after electroporation. To distinguish between s/v-Themis-B and s/v-Themis-B2, each locus was amplified using the primers that were used for genotyping listed in Table S2(c), and then the amplicon was used for the next PCR as a template. The target sites of each TALEN were amplified by PCR using primers listed in Table S2(d). Four hundred ng of DNA from the PCR was treated with phenol/chloroform and recovered by ethanol precipitation; it was then used for the SURVEYOR Mutation Detection Kit Cel-I assay according to the manufacturer’s instructions. Products were analysed by electrophoresis in 3% agarose gels with ethidium bromide staining as described previously^30^. The results are shown in Fig. S7.

### Fertilization experiment using animals mutagenized by TALENs

TALEN mRNAs were injected into embryos by electroporation. The injected embryos were cultured in a water tank until sexual maturation. Gravid animals were dissected for egg and sperm collection from the gonoduct and vas deference, respectively. Eggs were held in FSW for one hour at 20°C, and sperm were kept on ice until use. Nine hundred microliters of the egg-containing FSW was added to each well in a 24-well plate, and 100 μl of sperm diluted in FSW was added to each well so that the final numbers of sperm were 10^4^, 4 × 10^4^, or 10^3^. One hour after insemination, the number of cleaved eggs was counted, and the number was divided by the total number of eggs in each well to calculate the fertilization ratio (%). In the case of animals in which *s-Themis-A(A-2)* had been mutagenized by TALEN, the number of tadpole larvae was counted 18 hours after insemination to calculate the fertilization ratio (%) again.

## Supplementary information

Supplementary Information
