# Supplementary material for: Three multi-allelic gene pairs are responsible for self-sterility in the ascidian Ciona intestinalis
Source: Sci Rep. 2020 Feb 13;10:2514. doi: 10.1038/s41598-020-59147-4 (PMC7018956; doi:10.1038/s41598-020-59147-4)
Supplement: Supplementary file 1 — Supplementary Information [file 41598_2020_59147_MOESM1_ESM.pdf]

Supplementary information

Three multi-allelic gene pairs are responsible for self-sterility in the ascidian *Ciona intestinalis*

Hitoshi Sawada[1]* §, Kazunori Yamamoto[1] §, Akira Yamaguchi[1] §, Lixy Yamada[1], Arata Higuchi[1], Haruhiko Nukaya[1], Masashi Fukuoka[1], Tetsushi Sakuma[2], Takashi Yamamoto[2], Yasunori Sasakura[3], and Maki Shirae-Kurabayashi[1]

[1] Sugashima Marine Biological Laboratory, Graduate School of Science, Nagoya University, Sugashima, Toba 517-0004, Japan

[2] Department of Mathematical and Life Sciences, Graduate School of Science, Hiroshima University, Hiroshima 739-8526, Japan

[3] Shimoda Marine Research Center, University of Tsukuba, Shimoda, Shizuoka 415-0025, Japan

Table S1. A list of names and accession numbers of *s/v-Themis-A, -B,* and *-B2* alleles

(a) *s/v-Themis-A* alleles: names and sequences.

New name of *s/v-Themis-A* allele	Previous allele name	Satou's allele name	Accession No. of *v-Themis-A* cDNA	Accession No. of *s-Themis-A* cDNA	Accession No. or reference of gDNA
A-1	AP1	None	AB646592	AB646601	
A-2	G7	None	AB646596	AB646606	
A-3	H2	None	AB646599	AB646607	
A-4	JGI	None	AB646593	AB646609	
A-5	AP2	None	AB646594	AB646602	
A-6	BP1	None	AB646597	AB646603	
A-7	BP2	None	AB646595	AB646604	
A-8	DP	None	AB646598	AB646608	
A-9	H10	None	AB646600	AB646608	

(b) *s/v-Themis-B* alleles: names and sequences.

New name of *s/v-Themis-B* allele	Previous allele name	Satou's allele name	Accession No. of *v-Themis-B* cDNA	Accession No. of *s-Themis-B* cDNA	Accession No. or reference of gDNA
B-1	y1-7-B				LC425348
B-2	JGI		AB646587	AB646582	
B-3	82	D	AB646591	AB646586	Ref 17
B-4	177		AB646590	AB646585	
B-5	192		AB646589	AB646584	
B-6	JP1 or 02*				LC425349
B-7	96			LC425392	
B-8	i12		AB646588	AB646583	LC425350
B-10	01*				LC425352
B-11	03*	C			LC425353, Ref 17
B-12		B			Ref 17
B-13		E			Ref 17
B-14		G			Ref 17
B-15		J			Ref 17

* These alleles (named 1, 2 and 3) were cloned and sequenced by AH.
Note that the B-9/112 allele was previously assigned as a B-type allele but that the allele was re-examined and found to be a B2-type allele (B2-8).

(c) *s/v-Themis-B2* alleles: names and sequences.

New name of *s/v-Themis-B2* allele	Previous allele name*	Satou's allele name	Accession No. of *v-Themis-B2* cDNA	Accession No. of *s-Themis-B2* cDNA	Accession No. or reference of gDNA
B2-1	MI001	L			LC425354, Ref 17
B2-2	y1-7-B2				LC425355
B2-3	MI002	K			LC425356, Ref 17
B2-4	GM001	F			LC425357, Ref 17
B2-5	GM003	A			LC425358, Ref 17
B2-6	GM205				LC425359
B2-7		I			Ref 17
B2-8	112	H		LC425393	LC425351, Ref 17

The previous names of the respective haplotypes are indicated in the second column. GM and MI series individuals were obtained from Gamagori and Maizuru, respectively.

Table S2. Primers used in this study.

(a) Primers for amplification of *s/v-Themis-B* and *s/v-Themis-B2* from genomic DNA.

Name of primer	Region	Nucleotide sequence
P1(TBg)	KH.C7.499.v1.A.ND1-1 Fwd	5’-AAATTATTTCTGGTGACGCACGTTG-3’
P2(TBg)	KH.C7.666.v1.SL2-1 Rev	5’-ATGTTTTTAACTTTCTGCGGGAGGA-3’
P3(TB2g)	s-Themis-B2 conserved region Fwd	5’-ACCTCCAGCTTTCTCGTGGGAACCT-3’
P4(TB2g)	KH.C7.700.v1.C.ND1-1 Rev	5’-GTGGTACAGCAGTTCAACAACTGAGAGT-3’

(b) Primers for amplification of cDNA of *s/v-Themis-B*.

Name of primer	Region	Nucleotide sequence
P1(sTBc)	s-Themis-B conserved region Rev	5’-ACCTCCAGCTTTCTCGTGGGAACCT-3’
P2(sTBc)	s-Themis-B(082) Exon 1 Fwd	5’-CGCGGGGACAGAATATATTTCATGTTTG-3’
P3(sTBc)	s-Themis-B(096) Exon 1 Fwd	5’-ATTCATACAACCAAGTTGCTGAGGATGC-3’
P4(sTBc)	s-Themis-B(112) Exon 1 Fwd	5’-GCGGGGACAGATATAGGCCTACCAAT-3’
P5(sTBc)	s-Themis-B(177) Exon 1 Fwd	5’-TCCAAAGACCAGCAGTGACTGACTAGA-3’A
P6(sTBc)	s-Themis-B(192) Exon 1 Fwd	5’-GCGGGGACCACTACAACTATTTTTCTGA-3’
P7(vTBc)	v-Themis-B(082) Exon 1 Fwd	5’-CTATACCCTTGAACCAAGGTGTCCATATAA-3’
P8(vTBc)	v-Themis-B(177) Exon 1 Fwd	5’-CCAGTACACAGCAAATAAAAGAAATCTCAA-3’
P9(vTBc)	v-Themis-B(192) Exon 1 Fwd	5’-ATGAAGTGGCTGTGTATGATC-3’
P10(vTBc)	v-Themis-B(082) Exon 2 Rev	5’-TGTATTAATTAAGAACCAATGTCGCCAATA-3’
P11(vTBc)	v-Themis-B(177) Exon 2 Rev	5’-TGTATTAATAAAAGGCCAATAACGCCAGTA-3’
P12(vTBc)	v-Themis-B(192) Exon 2 Rev	5’-TTAACGATAATACTGTATCTTCAT-3’

(c) Primers for genotyping in an out-crossing experiment (Fig. S5).

Name of primer	Region	Usage	Nucleotide sequence
sTB/B2_common1	B, B2	Sequencing	5'-TTGGTTGGAACATTGGCATATAA-3'
7qrej5r3rev	B, B2	PCR	5'-ACCTCCAGCTTTCTCGTGGGAACCT-3'
Left-Themis-Upper-Fwd1	B	PCR, sequencing	5'-TATACTTGCCTTTGTGCCAACACAATAGTT-3'
KhC7-700_Rev1	B2	PCR, sequencing	5'-GTGGTACAGCAGTTCAACAACTGAGAGT-3'
C2.477-p1	A	PCR, sequencing	5'-ACAATGTAGTTTGTTAACGTTACAG-3'
sTAall-1-rev	A	PCR, sequencing	5'-TCACTTGGAATCAATTTACCGTT-3'

(d) Primers used for the Cel-I assay (Fig. S7).

Name of primer	Nucleotide sequence
Cel_I_sTB_No1_Fwd	5'-CCTGATGAATGTAAATTGGTTC-3'
Cel_I_sTB_No1_Rev	5'-GAATCAAACGGTAGACTTGCT-3'
Cel_I_sTA_No1_Fwd	5'-AACGGTAAATTGATTCCAAGTG-3'
Cel_I_sTA_No1_Rev	5'-TTCTGAATAACAAGCGGTGTG-3'
Cel_I_sTA_G7_Fwd	5'-AACCGACTTTAATATTACCAGG-3'
Cel_I_sTA_G7_Rev	5'-TTCTTTACCAAATGGGACGAG-3'

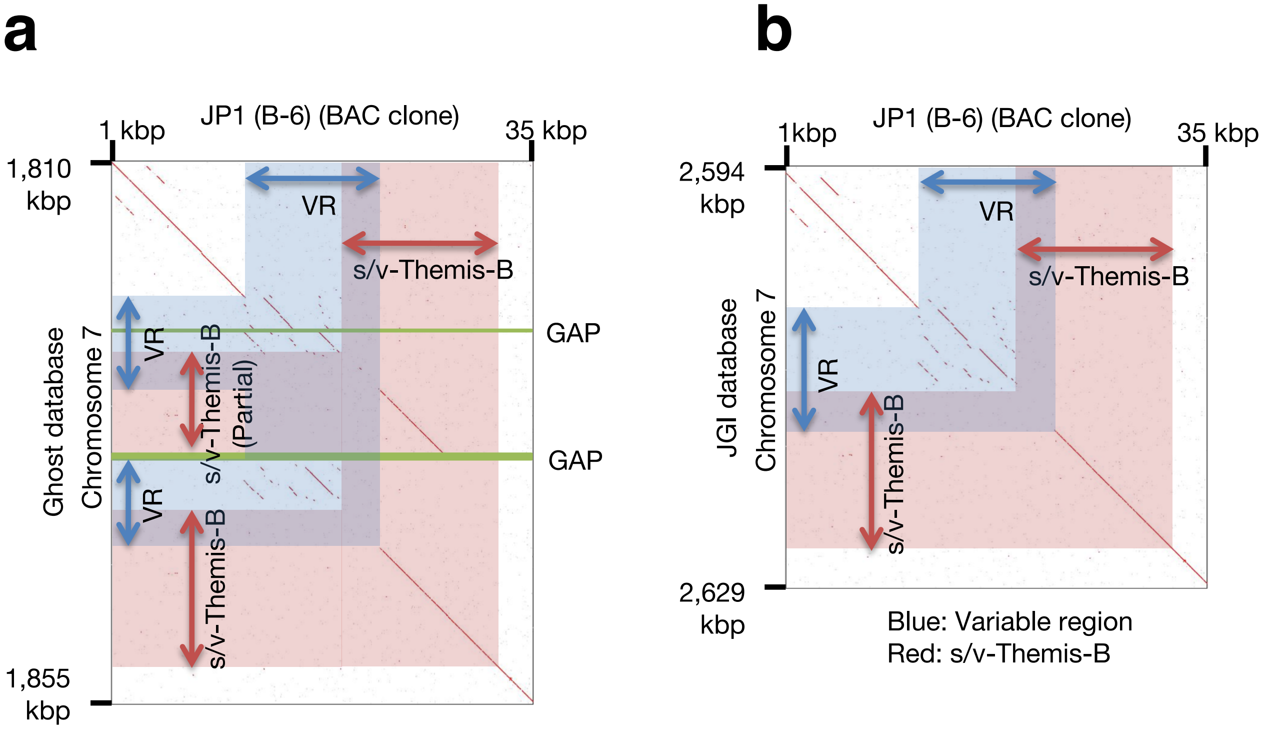

Fig. S1. **Dot plot analysis of the *s/v-Themis-B* region.**

An isolated clone of genomic DNA around the *s-Themis-B* region was compared with DNA sequences reported in the Ghost database (a) and JGI database (b). Note that the Ghost database contains two gaps. The JGI database sequence was identical to the sequence of the isolated clone. Red arrows indicate the region of *s/v-Themis-B,* and blue arrows indicate the variable region (VR) including the hyper-variable region (HVR) of *s/v-Themis-B.*

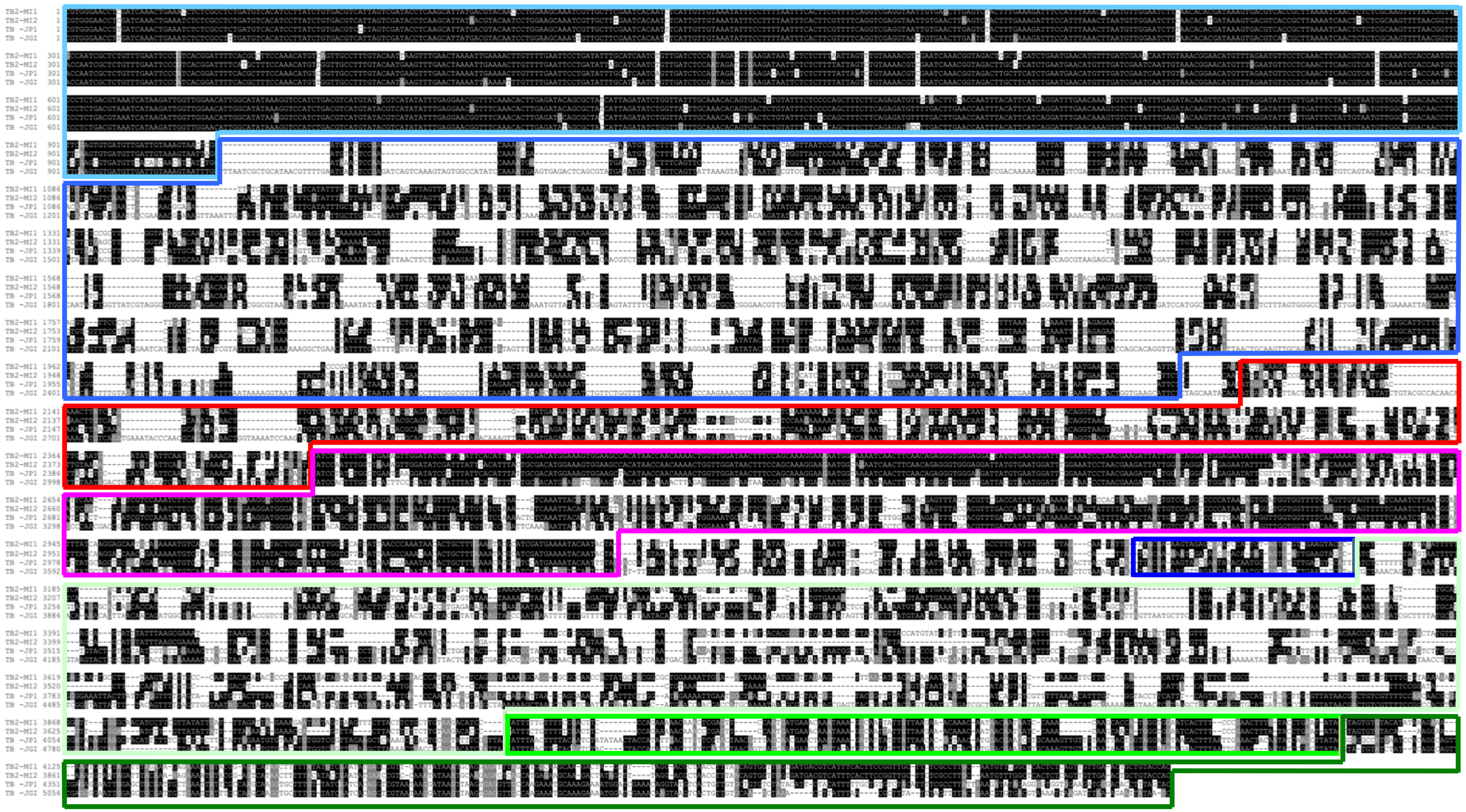

Fig. S2. **Comparison the DNA sequences of the *s/v-Themis-B* and *s/v-Themis-B2* genes.**

The determined nucleotide sequences of two *s/v-Themis-B2* alleles (B2-1 (=MI001) and B2-3 (=MI002)) and two *s/v-Themis-B* alleles (B-2 (=JGI) and B-6 (=JP1)) were aligned, and identical nucleotides are indicated by black highlighting. Genomic DNA regions are indicated by the following open, coloured boxes: sTB-CR (*s-Themis-B* conserved region: sky blue box), sTB-HVR2 (*s-Themis-B* hypervariable region 2: blue box), vTB-HVR (*v-Themis-B* hypervariable region: red box), vTB-WCR (*v-Themis-B* weakly conserved region: magenta box), sTB-HVR1 (*s-Themis-B* hypervariable region 1: dark blue box), sTB-USR1 (*s-Themis-B* upstream region 1: pale green box), sTB-USR2 (*s-Themis-B* upstream region 2: green box), and sTB-USR3 (*s-Themis-B* upstream region 3: dark green box).

a

```
vTA-BP1    1 -------MLVNMIFIFVVLFKVNGVLAYDN------TLSFTSSKYYEQ-TKISTIVRMLSNVKNLRQKQRTTCPIFR-SSMNEP-NTTSYTQHSGQDYTLLFKDCSEIYKAGSVKSDVYP
vTA-H10    1 -------MQVTICCMLLVLYEVKAIVAYGN------TLNNTKNGYWEQ-LRTTKVARKLLYIKDLQEKRKTNCLTARTSEWKQK-NSQINMGQELQHQTILFTDCNEIYKMGHANSDVYQ
vTA-DP     1 -------MPVSICCIVLVLLAVNLILAYNI------ETNVKINDFNKQPLKITKITRKVTNIKNIRQRQRTGCSILTADKLKQPENNVTGHNQQKNTITSLFQDCKEIYEAGYVRSGAYA
vTA-H2     1 -------MTDYICCIVLASLVVNLILANNI------ETNFKMNEFKEQ-LKITKIARMVTNIKNLRQEQKTGCSIITADELKQPEHNVTGYNQQKNTITSLFQDCKEIYDAGYVRSGAYA
vTA-AP2    1 ------MYGIVYYMLLIVLFQQNEIQAIDKKSQK---TNFTADD-YKQ-LKLTYVVRKLPSIKNFQQKQQTTCLGIRTKNQLKLQGDNTTRFKQIVRKTVLYQDCKAIYDAGYVESCVYP
vTA-BP2    1 ------MYGIVY-CILLVLFQQNKIQAIDNKSEQTITTNFTADDYYKQ-LKLIYIVRKLPNIKNFQQKLQTICPEIRTKNQLALQGGNTTRLTHIARKIVLYQDCKAIYDAGYVESDVYP
vTA-JGI    1 MARRKSMYRIVY-CILILLLEQNRIETLDSKSQRINTTNLTVDDYYKQ-LKLINIVRKLPNIKNFQQKQQTICSGICTTNQVNLQRDNATRLRQNARKIIVYQDCKAMYDAGYVESGVYP
vTA-G7     1 ------MYGIVFYSILIALFKQNLIQAFEKKSQQTITTNFTAGDHYKQ-LKLIYIVRKLQNVKNFQEKQQTTCSRIRTKHQLELQVDNTTRFKQIARNVVFYQDCKAMYDAGYVESGVYP
vTA-AP1    1 ------MRSIIY-CILFVLLENNRIVASKS------TPNFTTNNYHTQ-LQFINVVRKVSRTRNVQLKRKTTCSTVHKVNLVQLQVDNTAIRNQTVTRFLLFQDCNEIYKAGHLKSDVYP
                    .  ... ....... .. ... .       .....  ....* .... ..*.. ..........* *. ...    ....  .. .  .  . .....**...*..*.. * .*.

vTA-BP1  105 IWVNQLYKYTFVYCDMDVKAVSNKTGWIIIQRRINGEINFNRGWQNYVDGFGNVRGEHWVGLEHIHALSNQNTSIDQFGSYVTPPRMRIDFEDQEGVTAYAEYEQFKLVGANQKYRLIAA
vTA-H10  106 LWVNQLYRFTFVYCDMDVRAVRNQTGWITIQRRINGEINFDRGWKDYVDGFGNVRGEYWIGLEHIHALSNQNTTTNWFGAFVTLPRMRIDFEDQDGVTAYAEYELFKVAGANKKYRLIAV
vTA-DP   108 IWINRLYKFSEVFCDMDVQALSNKTGWITIQKRINGEINFDRGWQNYVDGFGNVRGEHWVGLENIHALTYQNTTNEWFGSYVTPPRMRIDFKDQEGVTAYAEYEVFKVAGIKDKYRLIAA
vTA-H2   107 IWINRLYKFTEVFCDMDVQALSNKTGWITIQKRINGEINFNRGWQNYVDGFGNVHEEHWVGLENIHALTYQNTTTEWFGTYVTSPRMRIDFVDQDGVTAYAEYEVFKVAGANKKYRLIAA
vTA-AP2  110 IWILKLYKFIHVYCDMDVQAVSNNTGWITVQRRINGEINFDRGWQNYVDGFGNVRGEHWLGLEHIHALTYQNTTTEWFGSYVTPPRMRVDFVDQEGVSAYAEYKLFEVSGAKQTYQLIAA
vTA-BP2  113 IWINKLYKFIHVYCDMDVKAVGNNTGWVTIQRRINGEINFDRGWQNYVDGFGNVHGEYWIGLENIHALTYQNTTTDQFGTYVTAPRMRVDFVDQDGVSAYAEYRLFKVLGAKQKYQLIAA
vTA-JGI  119 IWIYQLYKFTHVYCDMDVQAVGDKNGWITIQKRVNGAINFDRGWQNYVDGFGNVYGEYWIGLEHIHALTYQNTTTDQFGSYVTPPRMRIDFVDQDGVTAYAEYRVFVVSGAKQKYRLIAA
vTA-G7   114 IWIYKLYKFTHVYCDMDVQAVSNNTGWISIQKRVNGEINFDRGWQNYVDGFGNVRGEYWIGLKHIYALTNQNTTIDWIGSYVTPPRMRIDFVDQDGVSAYAEYKLFKVSGAEQKYRLIAA
vTA-AP1  107 IWLQQLYKLTYVFCDMDVQAVSNKTGWITIQKRVNGAINFDRGWQNYVDGFGNVRGEYWIGLENIHALSNQNTTTDWLGSYVTAPRMRIDLHDQDGISAYAEYKLFKVAEAKEKYRLIIA
             .*...**... *.*****.*.....**...*.*.**.***.***..********..*.*.**..*.**..***.....*..**.****.*..**.*..*****..*.. .....*.**..

vTA-BP1  225 YIDKATAIPLNVSSPISNYWFSTFDNNNAFSGCPRRFKGGWWFLACGKSNLNGQYPNKDEQMSSSNIYWYYWYIVNKNNTALKTVSMKFQY
vTA-H10  226 HLDKATAIPAGVPTPISTYWFSTYDNNNAYSNCPQTYHGGWWFLACGESNLNGPYPQIGDKNSPSNIYWYDWYTVNENNTAFKSVSMKFQY
vTA-DP   228 YLDQATAIPPGVPTPIAHHWFSTFDNNNALLGCPQTYHGGWWFLACSESNLNGPYPRMGDKNSPSNIYWYDWNTVNENNTAFKSVSMKFQY
vTA-H2   227 YLDQATAIPPGVPTPISNHWFSTYDNNNAFPGCPETFQSGWWFLACGESNLNGPYPQIGDKNSPSNIYWYDWYTVNENNTAFKSVSMKFQY
vTA-AP2  230 YLDQATAIPANAIAPIFRNRFSIYDNNNVFSNCPGKFYGGWWFSGCPRSNLNGLYPSQGEKNSPSNIYWYDWYTVNENNTAFKSVSMKFQY
vTA-BP2  233 YLDQATAIPPNAPSPIFRNWFSIYDNNYVFSNCHQILHGGWWFSACAKSNLNGPYPQMGDKVSPSNIYWYDWNTVNKNNTAFKSVSMS---
vTA-JGI  239 HLDQATAIPPNGSSPIYRVWFSIYDNNYVFSNCPEKLHGGWWFSGCPRSNLNGLYPTQGEKNSPSNIYWYDWYTVNENNTAFKSVSMKFQY
vTA-G7   234 HLDQATAIPRYVSSPISRNWFSTYDNNHAFSNCHETFHGGWWFSGCGESNLNGPYPQTGDKNSPSNIYWYDWYTVNENNTAFKSVSMKFQY
vTA-AP1  227 QLNQATAIPSNVPSPITRIWFSTFDND-DQKKCPEIFHSGWWFFACGESNLNGLYPKEGDKNSPSNIFWYYWYTVNENNTAFRSVSMKFQY
               ....*****  ....**.. .**..**......*.   ...****  .*..*****.**.  ....*.***.**.*..**.****...***....
```

b

Protein	Residues	Sequence	Mass (Theo-retical)	Mass (Experi-mental)
v-Themis-A (A-1 / AP1)	85-96	FLLFQDCNEIYK	1588.76	1588.85
	102-114	SDVYPIWLQQLYK	1651.86	1651.94
	149-161	GWQNYVDGFGNVR	1510.70	1510.79
	223-244	LIIAQLNQATAIPSNVPSPITR	2316.32	2316.40
v-Themis-A (A-2 / G7)	161-173	GWQNYVDGFGNVR	1510.70	1517.79
	235-248	LIAAHLDQATAIPR	1488.84	1488.89
v-Themis-A (A-3 / H2)	58-69	TGCSIITADELK	1306.64	1306.70
	83-93	NTITSLFQDCK	1325.63	1325.70
	103-112	SGAYAIWINR	1149.59	1149.65
v-Themis-A (A-4 / JGI)	122-130	KIIVYQDCK	1165.62	1165.56
	233-248	IDFVDQDGVTAYAEYR	1860.85	1860.80
	313-325	SNLNGLYPTQGEK	1420.68	1420.76
v-Themis-A (A-5 / AP2)	155-163	INGEINFDR	1077.51	1077.56
	164-174	GWQNYVDGFGNVR	1510.70	1510.79
v-Themis-A (A-6 / BP1)	159-171	GWQNYVDGFGNVR	1510.70	1510.79
v-Themis-A (A-7 / BP2)	146-154	INGEINFDR	1077.51	1077.56
	227-250	YQLIAAYLDQATAIPPNAPSPIFR	2629.39	2629.46
v-Themis-A (A-8 / DP)	74-83	TGCSILTADK	1064.52	1064.56
	99-109	NTITSLFQDCK	1325.63	1325.70
	112-128	SGAYAIWINR	1149.59	1149.65
	157-165	INGEINFDR	1077.51	1077.56
	166-178	GWQNYVDGFGNVR	1510.70	1510.79
v-Themis-A (A-9 / H10)	139-147	INGEINFDR	1077.51	1077.56
	148-160	GWKDYVDGFGNVR	1511.72	1511.76

Fig. S3. Proteomic analysis of v-Themis-A.

(a, b) Trypsin-digested fragments of nine v-Themis-A alleles were detected by proteomic analysis of the VC proteins using the MASCOT search engine. Theoretical and experimental mass values are indicated. Peptides with scores higher than 20 were accepted as identified. The sequences indicated by blue or red indicate the same sequences, suggesting the occurrence of a relatively conserved region in v-Themis-A. Positions of respective peptides in (b) are indicated in (a).

a

```
vTB-082    1 MKWIYFQVVFS-----LYMAIPLNQGVHITATGFQIKVINLSRQLLLCRELIGLRNEKCKN---ILEKEIRKLKVSLFEVG-AKRNALTAAQNLNTDRTNIHLSGKYVSDVYPIWLKVGY
vTB-192    1 MKWLCMILILKIAP--LFINGGIETGIQIEKS-LQKKVINLTKQLLHCRDLIHVRKNRCRVTGDILVKEIHLVIRRLVPNSDSTLKKVSAIRSLYTDCNYVLQTGRYVSEVYPIWLKVGY
vTB-177    1 MKSYTLFAFLVFKT--LLCKTSTQQ--IKEISIMQTEVAKLPKQVQFVKQLMAPRMLTCQDFRLSFNTSVYSISHQYMIDA--KIEEYSPIYQMFTECKSIWFSGNLKSGIYPIWLLSQY
vTB-JGI    1 MKTLLILVVIICTPQQNMSDEIPNADKTGKIQVVRNDMIIAVDALNKVVHEMKRRLEMCQK-----NKNLFQLEKSSQNVS---TLYQQPILKLYKDCTSIYTANFVSNGIYPLWLTSAY
             **.. ......   .   .  .    ..... ....... ........... .... .*...*.    ......  . .... ..   ..   .. . .......... ........**.**. .*

vTB-082  112 RFIDIYCDMESGSYITNKTGWITFQQRKNGKINFNRGWEDYVNGFGNPNEDYWAGLENILMLTNQNLIVLNHG-YSTLRPNLRVDLKGWDGFNAHVEYERFTLYSNRN-YKIGRFGKRYG
vTB-192  118 RFIHIYCDMESGSHITNKTGWKTFQQRKNGKINFNRGWDDYVNGFGNPNEDYWAGLENTYLLTRQNEIGFSYG-YRTTRPNLRIDLEGWDGFHAYVEYKPFSINPEEN-YSISGLGVRFG
vTB-177  115 RYIYVQCDMDKRSEIGNKTGWVVFQQRQDGTINFNRGWESYVSGFGNLNNDYWAGLENIMLLTIQKTAVSRFG-YNTIFPNLRVDMEGWDGFHAYVEYEDFFIYTKKYRYEISDLGKRYG
vTB-JGI  113 QFIHVFCNMKVKSNITKLRSWAIIQRRINGDINFHRGWVDYVNGFGNPNEEHWMGLEIIERLTQQRITYKPYGRPTTDFPRLRIDLEDWDGFTAFVEYSKFLLLPDSKNYKFSTIGTVFG
             ..*...*.*...* *.....*...*.*..*.***.***..**.****.*...*.***....**.*....   .* ..*. *.**.*...****.*.***..*....... *... .*...*

vTB-082  230 TGFQPNFPDAFFRQEFSTFDKKSEGTSFQTNCPGPHNSGWWFSECGFSNLNGVYQGPNQKMSKRDGIYWRHWFLINTNKTALKHASMKTQYPFRGN
vTB-192  236 TAFHPDYRGSILPNDFSTFDHTSETSFFISNCPGPHNSGWWFFGCGYSNLNGIYQRPTDKMSKSDGIYWRHWTLINPNNTALKLASMKIQY-YR--
vTB-177  234 TAFASFTPDAFLKQEFSTFDNKDELKWFDTNCPGPLNGGWWFATCSLANLNGRYPHSFEKMSIHN-IYWRYWPFINTNNTALRYVSMKML------
vTB-JGI  233 NPDNHFFSREISSTGFSTFDHKTN-TFLNARCPAPDNGGWWLTSCSRSNLNGKYATSTERMSINN-MYWKLWFIVNPNYAALKHVSLMVQF-----
             .......... ....*****.... ......**.*.* ***...* ..**** *.. ...** . ..**..*...* *..**.. *..... ..
```

b

Protein	Residues	Sequence	Mass (Theo-retical)	Mass (Experi-mental)
v-Themis-B (B-2 / JGI)	228-245	FSTIGTVFGNPDNHFFSR	2041.96	2042.01
	246-258	EISSTGFSTFDHK	1454.67	1454.72
v-Themis-B (B-3 / 82)	98-108	YVSDVYPIWLK	1381.73	1381.77
	198-210	GWDGFNAHVEYER	1578.69	1578.76
	228-242	YGTGFQPNFPDAFFR	1762.81	1762.87
v-Themis-B (B-4 / 177)	103-115	SGIYPIWLLSQYR	1594.85	1594.93
	142-150	QDGTOMFNR	1063.50	1063.57
	186-196	FGYNTIFPNLR	1340.69	1340.74
	232-246	YGTAFASFTPDAFLK	1634.80	1634.86
v-Themis-B (B-5 / 192)	90-103	SLYTDCNYVLQTGR	1688.78	1688.86
	104-114	YVSEVYPIWLK	1395.78	1395.79

Fig. S4. Proteomic analysis of v-Themis-B

(a, b) Trypsin-digested fragments of four v-Themis-B alleles were detected by proteomic analysis of the VC proteins. As shown in Fig. S3, theoretical and experimental mass values are indicated. Peptides with scores higher than 20 were accepted as identified. Positions of peptides in (b) are indicated in (a).

Trial 1

a

Sperm

Egg

Trial 1	1	2	3	4	5	6	7	8	9	10	WT
1	2 (3/132)	2 (4/113)	94 (117/124)	100 (116/116)	98 (125/127)	NA	NA	NA	0 (0/116)	100 (149/151)	100
2	1 (1/119)	2 (2/88)	100 (89/89)	99 (66/67)	100 (36/36)	NA	NA	NA	NA	NA	100
3	100 (203/203)	100 (191/191)	1 (1/173)	100 (188/188)	100 (166/166)	NA	NA	NA	100 (161/161)	100 (173/173)	100
4	0 (0/320)	1 (3/345)	1 (3/307)	0 (1/310)	0 (1/430)	0 (0/365)	NA	0 (0/365)	NA	NA	100
5	100 (138/138)	25 (49/193)	17 (36/216)	0 (1/141)	2 (4/161)	32 (48/150)	NA	2 (2/123)	NA	NA	100
6	NA	NA	NA	1 (1/113)	8 (12/142)	0 (1/148)	4 (6/162)	NA	NA	NA	100
7	NA	NA	NA	NA	NA	0 (0/236)	1 (2/210)	NA	NA	NA	100
8	NA	NA	NA	6 (15/248)	3 (10/319)	NA	NA	5 (11/205)	2 (4/209)	99 (196/198)	100
9	36 (59/163)	27 (19/164)	100 (179/180)	NA	NA	NA	NA	100 (162/162)	0 (0/158)	100 (169/170)	100
10	35 (47/133)	10 (30/300)	100 (396/396)	NA	NA	NA	NA	100 (356/357)	0 (0/361)	0 (0/348)	100
WT	100	100	100	100	100	100	100	100	100	100	NA

- Two or more haplotypes mismatched
- B and B2 (but not A) haplotypes matched
- A and B2 (but not B) haplotypes matched
- A and B (but not B2) haplotypes matched
- A, B and B2 haplotypes matched

b

Trial 1	A	B	B2
1	A−1/A−2	B−1 homo	B2−1/B2−3
2	A−1/A−2	B−1 homo	B2−1 homo
3	A−1/A−2	B−2 homo	B2−2 homo
4	A−1/A−2	B−1/B−2	B2−1/B2−2
5	A−1 homo	B−1/B−2	B2−1/B2−2
6	A−1/A−2	B−1/B−2	B2−1/B2−2
7	A−1/A−2	B−1/B−2	B2−1/B2−2
8	A−1 homo	B−1/B−2	B2−1/B2−2
9	A−1 homo	B−1 homo	B2−1 homo
10	A−1 homo	B−1/B−2	B2−1/B2−3

Trial 2

a

Sperm (columns) / Egg (rows)

Trial 2	1	2	3	4	5	6	7	8	9	WT
1	0 (0/93)	81 (64/79)	84 (76/91)	22 (20/93)	100 (80/80)	100 (81/81)	38 (40/105)	6 (8/128)	100 (74/74)	NA
2	86 (83/97)	1 (1/90)	0 (0/88)	83 (85/102)	100 (79/79)	100 (84/84)	1 (1/93)	40 (16/40)	100 (66/66)	NA
3	100 (96/96)	6 (5/88)	0 (0/52)	98 (65/66)	100 (76/76)	100 (66/66)	36 (26/72)	100 (47/47)	100 (46/46)	NA
4	98 (100/102)	100 (40/40)	93 (85/91)	0 (0/51)	100 (38/38)	100 (56/56)	100 (37/37)	3 (1/39)	98 (44/45)	NA
5	93 (54/58)	90 (64/71)	100 (81/81)	100 (80/80)	0 (0/61)	21 (20/97)	91 (106/116)	100 (42/42)	100 (45/45)	NA
6	86 (31/36)	100 (54/54)	100 (19/19)	100 (16/16)	4 (4/90)	2 (1/41)	100 (44/44)	100 (8/8)	100 (10/10)	NA
7	88 (76/86)	1 (1/98)	1 (1/88)	93 (89/96)	100 (94/94)	100 (62/62)	0 (0/102)	75 (52/69)	100 (52/52)	NA
8	14 (13/93)	94 (16/17)	100 (9/9)	NA	NA	NA	71 (67/94)	0 (0/102)	NA	NA
9	100 (83/83)	100 (102/102)	97 (76/78)	0 (0/84)	88 (44/50)	100 (40/40)	100 (47/47)	0 (0/74)	0 (0/49)	NA
WT	100 (85/85)	100 (92/92)	100 (88/88)	100 (95/95)	100 (83/83)	100 (98/98)	100 (85/85)	100 (102/102)	100 (105/105)	0 (0/103)

- Green: Two or more haplotypes mismatched
- Orange: B and B2 (but not A) haplotypes matched
- Cyan: A and B2 (but not B) haplotypes matched
- Yellow: A and B (but not B2) haplotypes matched
- Red: A, B and B2 haplotypes matched

b

Trial 2	A	B	B2
1	A−1/A−3	B−1 homo	B2−1/B2−2
2	A−2/A−3	B−1 homo	B2−1 homo
3	A−2/A−3	B−1 homo	B2−1 homo
4	A−1/A−2	B−1 homo	B2−1 homo
5	A−2/A−3	B−1/B−2	B2−2 homo
6	A−1/A−3	B−1/B−2	B2−2 homo
7	A−2/A−3	B−1 homo	B2−1 homo
8	A−1/A−2	B−1 homo	B2−1 homo
9	A−1/A−2	B−1/B−2	B2−1/B2−2

Trial 3

a

Sperm

Egg

Trial 3	1	2	3	4	5	6	7	WT	WT2
1	0 (0/98)	100 (94/94)	0 (0/108)	3 (3/103)	0 (0/95)	90 (75/83)	5 (6/116)	31 (26/85)	100
2	98 (81/83)	0 (0/82)	93 (83/89)	62 (87/140)	99 (86/87)	17 (25/156)	100 (94/94)	26 (28/107)	100
3	30 (54/178)	99 (102/103)	0 (0/89)	100 (83/83)	0 (0/104)	100 (88/88)	5 (7/128)	2 (4/162)	100
4	0 (2/115)	73 (119/164)	2 (2/103)	2 (3/124)	1 (1/101)	3 (4/117)	91 (94/103)	64 (67/105)	100
5	53 (55/104)	71 (42/59)	9 (10/112)	47 (36/76)	10 (6/63)	100 (46/46)	2 (1/55)	53 (25/47)	100
6	96 (86/90)	47 (52/110)	92 (77/84)	97 (86/89)	98 (94/96)	0 (0/92)	100 (111/111)	13 (9/71)	100
7	61 (41/67)	70 (43/61)	0 (0/54)	100 (41/41)	0 (0/34)	100 (34/34)	0 (0/32)	5 (1/22)	100
WT	100 (114/114)	90 (80/89)	94 (102/109)	100 (76/76)	100 (96/96)	99 (88/89)	99 (84/85)	0 (0/81)	100

- Two or more haplotypes mismatched (green)
- B and B2 (but not A) haplotypes matched (orange)
- A and B2 (but not B) haplotypes matched (cyan)
- A and B (but not B2) haplotypes matched (yellow)
- A, B and B2 haplotypes matched (red)

b

Trial 3	A	B	B2
1	A−1/A−2	B−1/B−2	B2−2 homo
2	A−2/A−3	B−1 homo	B2−1 homo
3	A−1 homo	B−1/B−2	B2−2 homo
4	A−1/A−2	B−1/B−2	B2−1/B2−2
5	A−1 homo	B−1/B−2	B2−2 homo
6	A−1/A−3	B−1 homo	B2−1 homo
7	A−1/A−3	B−1/B−2	B2−2 homo

Fig. S5. Raw data of fertilization ratios in crossing with selfed F1 and F2 siblings.

(a) Upper numbers indicate % of fertilization. Boxed highlight colours: orange (A haplotype mismatched); blue (B haplotype mismatched); yellow (B2 haplotype mismatched); green (two or three haplotypes mismatched); and red (all three haplotypes matched). (b) Haplotypes of A, B and B2 in each individual were identified by PCR using allele-specific primers, and the results are summarized.

a Exon/Intron organization of *s/v-Themis-B2 (B2-3/MI002)*

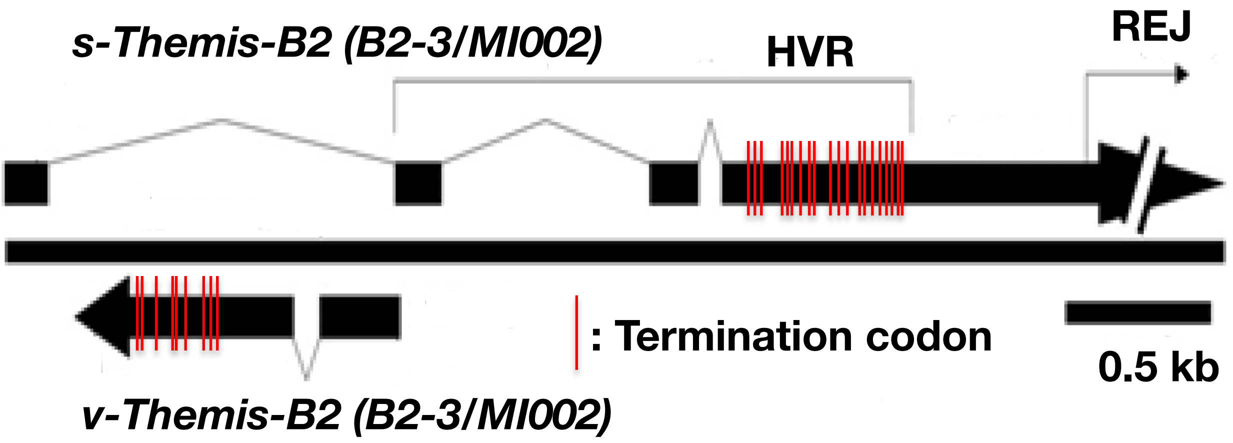

b *s-Themis-B2 (B2-3/MI002)* (20 termination codons)

```
ATG CTA TTA ATT CAA GCA CTG GCT TCG AAT AGT ACT GCA AGC GTA GTA GAA ATT AAT GCT CCG GTG GGC GAA GAA TGC AAC CGA GCG AAC GTT TCT AAT C  < 100
M   L   L   I   Q   A   L   A   S   N   S   T   A   S   V   V   E   I   N   A   P   V   G   E   E   C   N   R   A   N   V   S   N   Q
          10          20          30          40          50          60          70          80          90

AA ATT TCT CTC GAA TTT AAT TTA AAG GAA AAA CAG ATG CTT GTT CTT CGT TGG AAA AAT GGA AGC TGC AAC GTT GTT TCT CTT GTT GTA CGA TTG GAA CA  < 200
    I   S   L   E   F   N   L   K   E   K   Q   M   L   V   L   R   W   K   N   G   S   C   N   V   V   S   L   V   V   R   L   E   Q
          110         120         130         140         150         160         170         180         190

G GAA ACG ACA GTA TTT CCA TGT AAT GAA TCG CCA ATG AAG GAA CAA CTT TAC TTA ACC CAC AAA ATT TCT GTA TTT ATA TCT CCA CAA CCA GAA AAT TGC  < 300
   E   T   T   V   F   P   C   N   E   S   P   M   K   E   Q   L   Y   L   T   H   K   I   S   V   F   I   S   P   Q   P   E   N   C
          210         220         230         240         250         260         270         280         290

AAA CCA AAT GTC ATA GCA AGC TAT GTC ATA CTA GAA ATG GCT CTC ACT TTA CCA ATC AAA TCG GTT TCT ACT AGA GAA AAT ATA ACA ATC TAT GCT GAT A  < 400
K   P   N   V   I   A   S   Y   V   I   L   E   M   A   L   T   L   P   I   K   S   V   S   T   R   E   N   I   T   I   Y   A   D   I
          310         320         330         340         350         360         370         380         390

TT AGT AAC ATC AGA CAA TAC ACT GAA AAA TTC CTT ACA ACC ATT AGC TGT TCT ATT GCC TTT GGT AAC GGT TTG TCT GAA TCA ATT GGA ATT CGT CAA AA  < 500
    S   N   I   R   Q   Y   T   E   K   F   L   T   T   I   S   C   S   I   A   F   G   N   G   L   S   E   S   I   G   I   R   Q   K
          410         420         430         440         450         460         470         480         490

A TGT AAT TTA TCA TCG ATT TTT CGT GCC TGG TAA ACA GGA AAT ATC CTT TTC CTG TTA TGT ACC AAG CTC AAA AGA AAT TAG AGA GCA AAA AAG TAT ACA  < 600
   C   N   L   S   S   I   F   R   A   W   *   T   G   N   I   L   F   L   L   C   T   K   L   K   R   N   *   R   A   K   K   Y   T
          510         520         530         540         550         560         570         580         590

AGT GGA AAG TTT GTT GAA TGG TAA TAC CCT GAA AAT AAA ATC GGC ATC GGA ACC GGT CAG GTT CCT AAC GGA TAC CAA TCT GCT CTT CCA GCA TGT GTA C  < 700
S   G   K   F   V   E   W   *   Y   P   E   N   K   I   G   I   G   T   G   Q   V   P   N   G   Y   Q   S   A   L   P   A   C   V   P
          610         620         630         640         650         660         670         680         690

CA ATA TCC TGC AAT ACT GTG CTT CCT TTT AAA AGA TTA TAA AAG CTG TGA CAC AAC TAA AGA TTT CTT ACC AAC TAA ATA CGA AAA CAA AGA GGT TGT TG  < 800
    I   S   C   N   T   V   L   P   F   K   R   L   *   K   L   *   H   N   *   R   F   L   T   N   *   I   R   K   Q   R   G   C   *
          710         720         730         740         750         760         770         780         790

A GGT TCA GTT TGA ACT AAC AAC AAT CAT TCA ATC TAC AAT TGG ACC TGG AAT ACA TCC ATT TGC ATT ATA CTT CCA AAA TAA TGT TTC TGC AGT GCA TTA  < 900
   G   S   V   *   T   N   N   N   H   S   I   Y   N   W   T   W   N   T   S   I   C   I   I   L   P   K   *   C   F   C   S   A   L
          810         820         830         840         850         860         870         880         890

TAG GTC TGT GAT TTG GAT TAA CCA ACA AGT AAC TGG AAT AAA AGT CAC ATG CGA TCC CTT TGT TGG AAT ACA TCC TAA TTA CTT TAC AAT CAA CAT CAC A  < 1000
*   V   C   D   L   D   *   P   T   S   N   W   N   K   S   H   M   R   S   L   C   W   N   T   S   *   L   L   Y   N   Q   H   H   T
          910         920         930         940         950         960         970         980         990

CT TAA AGA AAG TTG TCC AGC CAA CAT CAC AGT AGA AAT CAA TAA ATA TCG CAA TAA TGA ACT TGT ATC TCA AAT GAA GAC ACT TTG TTC AAA TCC TGA TG  < 1100
    *   R   K   L   S   S   Q   H   H   S   R   N   Q   *   I   S   Q   *   *   T   C   I   S   N   E   D   T   L   F   K   S   *   *
          1010        1020        1030        1040        1050        1060        1070        1080        1090

A ATG TAA ATT GGT TCA AGT CAA AAC ATC TCT GAA GAA  < 1137
   M   *   I   G   S   S   Q   N   I   S   E   E
          1110        1120        1130
```

c *v-Themis-B2 (B2-3/MI002)* (9 termination codons)

```
ATG TTG TTA TTA GAA GAA TCG TTC AAA CTT ATA GGT GGC TTA CCT TCC AAG AAA AAC AGT TTC GAA CTG TTC AAC TCG TTG GCA ATT CAA AAC TTT TAC G  < 100
M   L   L   L   E   E   S   F   K   L   I   G   G   L   P   S   K   K   N   S   F   E   L   F   N   S   L   A   I   Q   N   F   Y   A
          10          20          30          40          50          60          70          80          90

CA GAT TGT AAA AGT GTT ATT CNN TCC GAT AAA AAT GGC AGC ATT TAT CCA ATT TGG TTA AAA GTT GGA TAT CGA TTT ATT TAC ATT TAC TGC GAC ATG GA  < 200
    D   C   K   S   V   I   X   S   D   K   N   G   S   I   Y   P   I   W   L   K   V   G   Y   R   F   I   Y   I   Y   C   D   M   E
          110         120         130         140         150         160         170         180         190

A AGT GGA AGC CAC ATA ACC AAC AAA ACT GGT TGG ATA ACT TTC CAG CAA AGG AAG AAT GGC AGA ATC AAC TTC AAC AGA GGG TGG GAG GAT TAT GTG AAT  < 300
   S   G   S   H   I   T   N   K   T   G   W   I   T   F   Q   Q   R   K   N   G   R   I   N   F   N   R   G   W   E   D   Y   V   N
          210         220         230         240         250         260         270         280         290

GGA TTT GGA AAT CCT AAC GAA GAT TAT TGG GCA GGA CTC GAG AAT ATT TTA TCG CTG ACC AGA CAA AAC GAA ATT GCA TAT AAA TAC GGC TAC TCG GTC C  < 400
G   F   G   N   P   N   E   D   Y   W   A   G   L   E   N   I   L   S   L   T   R   Q   N   E   I   A   Y   K   Y   G   Y   S   V   R
          310         320         330         340         350         360         370         380         390

GT CGA CCC AAT CTT CGT GTT GAT TTG GAA GGA TGG GAC GGT TTC CAT GCG TAT GTA GAG TAT AAA ATG TTT ACG TTG TAT TCA AAA TCA AAT TAC ACG AT  < 500
    R   P   N   L   R   V   D   L   E   G   W   D   G   F   H   A   Y   V   E   Y   K   M   F   T   L   Y   S   K   S   N   Y   T   I
          410         420         430         440         450         460         470         480         490

T GTT AAT GTA GGG ACT CGT TTC GGA ACA GCA TTT CAA CCA AAA TAT TCG CAC GCG ATT TTT TTC GCT AAA GTT TAC CAC GTT TGA CCA CAT TAG TGA AAA  < 600
   V   N   V   G   T   R   F   G   T   A   F   Q   P   K   Y   S   H   A   I   F   F   A   K   V   Y   H   V   *   P   H   *   *   K
          510         520         530         540         550         560         570         580         590

AGC CTA TTT TAT TTC TAA TTG TCC AGG TCC CCA TAA TAG CGG GTG GTG GTT TTC GGG TTG CAG TTT TTC AAA TCT TAA CGG CGT TTA TCA AGG ACC AAC C  < 700
S   L   F   Y   F   *   L   S   R   S   P   *   *   R   V   V   V   F   G   L   Q   F   F   K   S   *   R   R   L   S   R   T   N   R
          610         620         630         640         650         660         670         680         690

GA AAA AAT GTC TAA ACG TGA CGG TAT ATA CTG GGG TCA CTG GTC TTT AAT TTT TCC  < 756
    K   N   V   *   T   *   R   Y   I   L   G   S   L   V   F   N   F   S
          710         720         730         740         750
```

Fig. S6. **B2-3/MI002 haplotype of *s/v-Themis-B2* is a pseudogene possessing several termination codons in respective open reading frames (ORFs).**

cDNAs of B2-3/MI002 haplotypes of *v-Themis-B2* were cloned from ovary mRNA by RT-PCR. The primers used for *v-Themis-B2* are vTB2_MI002_ORF_F: ATGTATTTGCTTTGTACATCGTTGG and vTB2_MI002_ORF_R: TTAGTATTGTATTTTCATCGATGCG. cDNAs of B2-3/MI002 haplotypes of *s-Themis-B2* were cloned from testis mRNA using a SMARTer RACE 5'/3' Kit, and the DNA sequences were determined. The primers for *s-Themis-B2* are sTBC_common1: TTGGTTGGAACATTGGCATATAA and Ci-sTB_5RACE2: TACCAAGTTGTCGCTTGTTGCCTCC. The results showed that *s-Themis-B2* and *v-Themis-B2* (B2-3 allele) cDNAs had 20 and 9 termination codons in the ORF, respectively. (a) Schematic representation of the positions of termination codons in ORFs of *s-Themis-B2* (B2-3) and *v-Themis-B2* (B2-3). (b, c) Nucleotide and deduced amino acid sequences of *s-Themis-B2* (B2-3 allele) (b) and *v-Themis-B2* (B2-3 allele) (c).

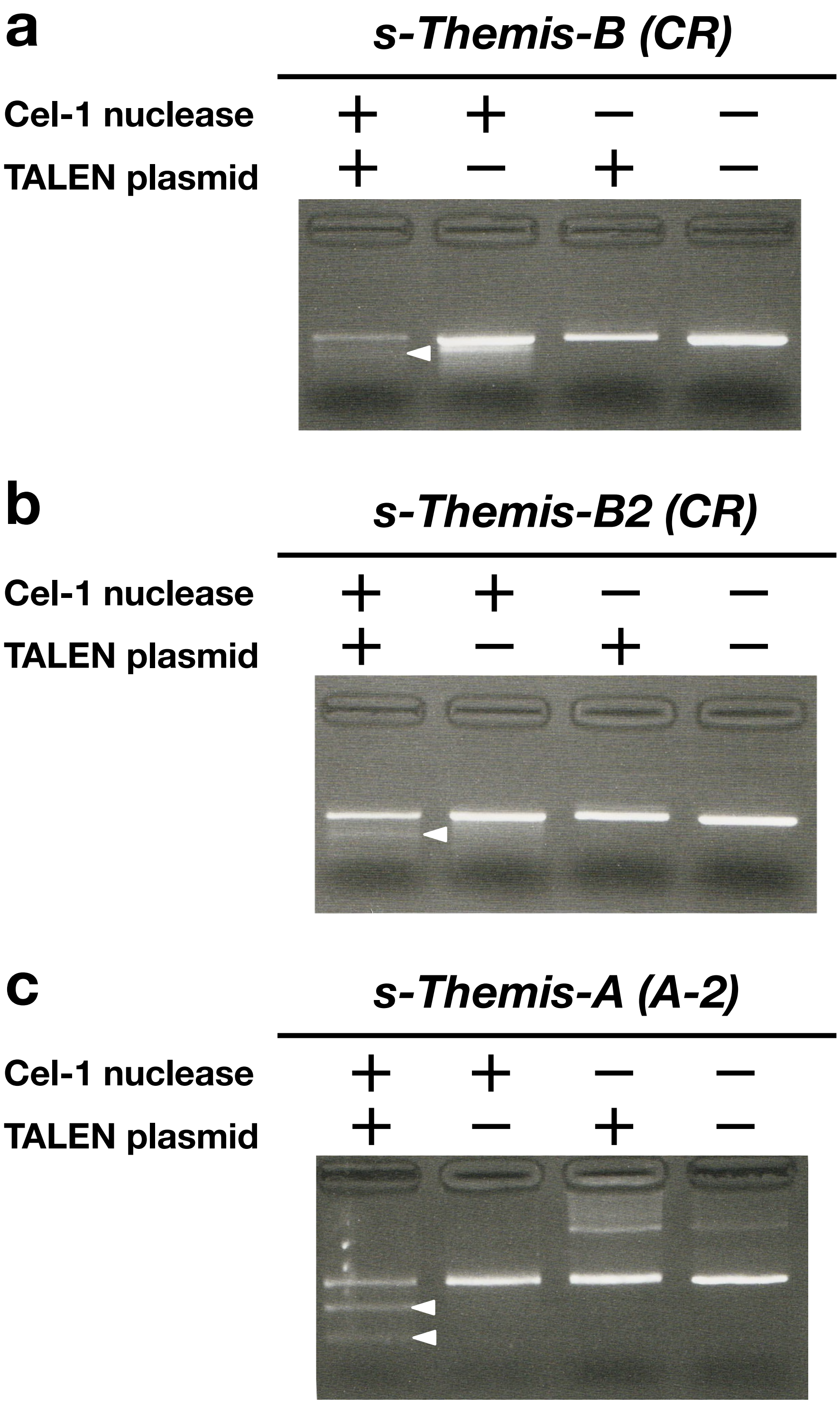

Fig. S7. TALEN induces mutations in targeting sites of the *s-Themis-A, -B and -B2* genes.

Mutations were induced by *s-Themis-B* (a)*, -B2* (b) *and -A* (c) TALENs, as revealed by Surveyor (Cel-I) nuclease. The primers used in the Cel-I assay are listed in Table S2 (d). Arrowheads indicate the positions of the Cel-I-digested bands. The presence of Cel-I-digested bands suggests the introduction of mutations.

a *s-Themis-B/B2 (CR)* No.1

Batch	B	B2
1	—	—
2	—	9bp del
3	—	9bp del
4	—	9bp del
5	—	9bp del
6	—	9bp del
7	—	9bp del
8	—	—
9	—	3bp del
10	—	11bp del
11	—	—
12	—	—
13	—	—
14	2bp del	—
15	—	3bp del
16	—	3bp del
total	1	10

b *s-Themis-B/B2 (CR)* No.4

Batch	B	B2
1	—	—
2	—	7bp del
3	—	—
4	—	—
5	—	—
6	6bp del	—
7	—	—
8	2bp del	6bp del
9	—	—
10	—	—
11	—	—
12	—	—
total	2	2

c *s-Themis-A (A-2)* No.3

Batch	
1	—
2	—
3	—
4	—
5	—
6	—
7	—
8	—
total	0

d *s-Themis-A (A-2)* No.7

Batch	
1	—
2	—
3	—
4	—
5	—
6	—
7	—
8	—
total	0

Fig. S8. **Mode of deletion/insertion in tadpole larvae developed from self-fertilized eggs of genome-edited individuals.**

(a, b) Mode of mutation in the targeting site of tadpole larvae developed from self-fertilized eggs derived from *s-Themis-B/B2* conserved region-targeted individuals (individual No. 1 (a) and No. 4 (b)) or from s-Themis-A (A-2 allele)-targeted individuals (individual No. 3 (c) and No. 7 (d)). Note that self-fertilized larvae do not necessarily contain targeted *s-Themis-A, -B,* or *-B2* genes.

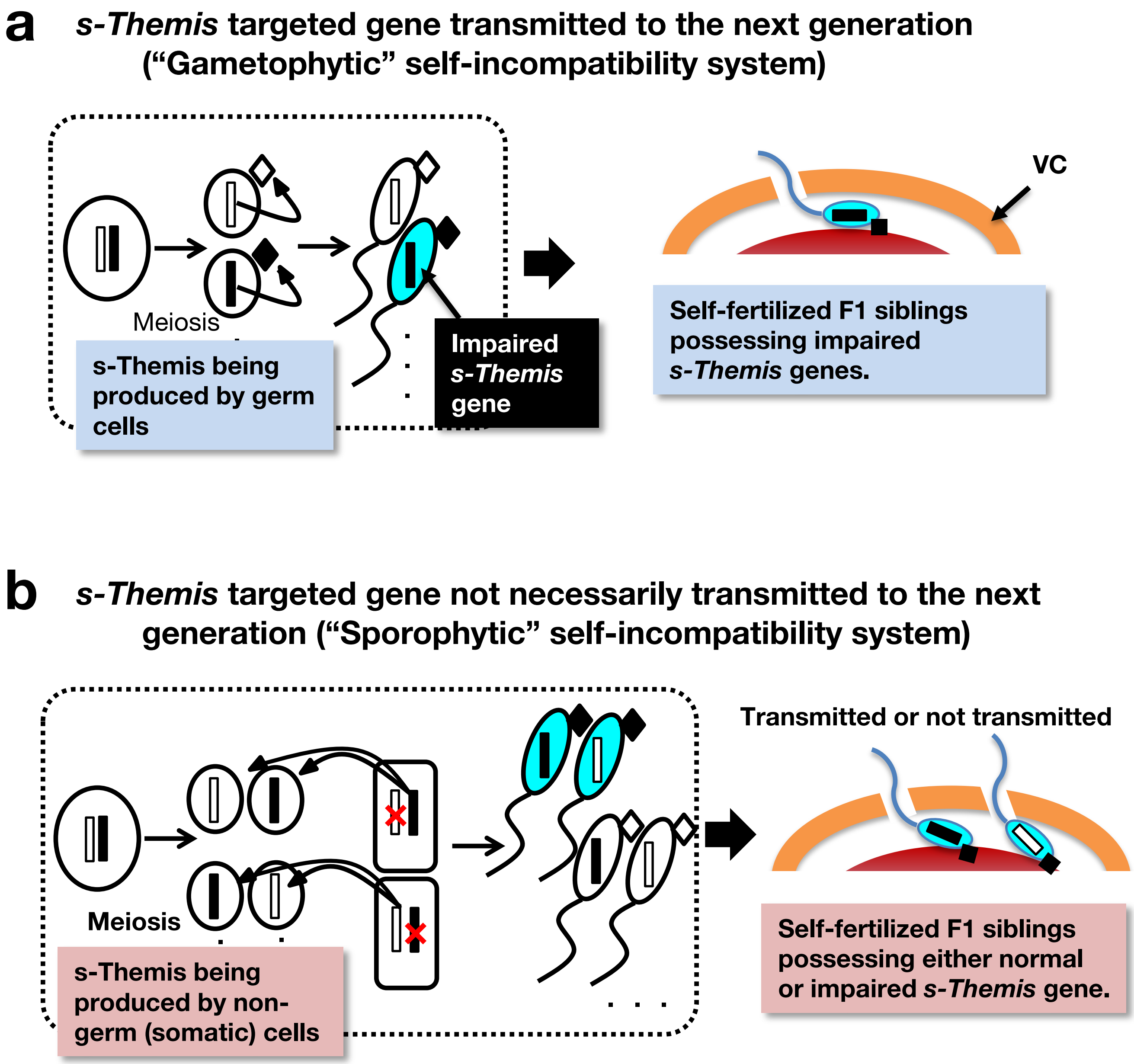

Fig. S9. Two possible expression modes of *s-Themis* genes considered by analogy to flowering plants: "gametophytic" and "sporophytic" self-incompatibilities.

There are two classical types of self-incompatibility systems in flowering plants: gametophytic and sporophytic. (a) If the mode of expression of the *s-Themis* gene is similar to the plant gametophytic self-incompatibility system, *s-Themis* genes are expressed by germ cells (haploid pollen in plants). In this case, targeted *s-Themis* genes will be transmitted to the next generation. (b) If the mode of expression of the *s-Themis* gene is similar to the plant "sporophytic" self-incompatibility system, *s-Themis* genes are expressed by non-germ cells (somatic cells). In this case, targeted *s-Themis* genes will not necessarily be transmitted to the next generation. Black rectangle, impaired s-Themis gene; black square, impaired s-Themis protein, blue-headed sperm, fertilizable sperm.
